# Granulocyte-Macrophage Colony-Stimulating Factor Modulates Myeloid-Derived Suppressor Cells and Treg Activity in Decompensated Cirrhotic Patients With Sepsis

**DOI:** 10.3389/fimmu.2022.828949

**Published:** 2022-06-03

**Authors:** Rashi Sehgal, Rakhi Maiwall, Vijayaraghavan Rajan, Mojahidul Islam, Sukriti Baweja, Navkiran Kaur, Guresh Kumar, Gayatri Ramakrishna, Shiv K. Sarin, Nirupma Trehanpati

**Affiliations:** ^1^ Department of Molecular and Cellular Medicine, Institute of Liver and Biliary Sciences, New Delhi, India; ^2^ Amity Institute of Biotechnology, Amity University, Noida, India; ^3^ Department of Hepatology, Institute of Liver and Biliary Sciences, New Delhi, India; ^4^ Department of Clinical Research and Biostatistics, Institute of Liver and Biliary Sciences, New Delhi, India

**Keywords:** myeloid-derived suppressor cells, sepsis, GM-CSF, Tregs, immune modulation, liver cirrhosis

## Abstract

**Background:**

Decompensated cirrhosis patients are more prone to bacterial infections. Myeloid-derived suppressor cells (MDSCs) expand in sepsis patients and disrupt immune cell functions. Granulocyte-macrophage colony-stimulating factor (GM-CSF) therapy helps in restoring immune cell functions and resolving infections. Its role in MDSC modulation in cirrhosis with sepsis is not well understood.

**Methods:**

A total of 164 decompensated cirrhotic—62 without (w/o), 72 with sepsis, and 30 with sepsis treated with GM-CSF—and 15 healthy were studied. High-dimensional flow cytometry was performed to analyze MDSCs, monocytes, neutrophils, CD4 T cells, and Tregs at admission and on days 3 and day 7. *Ex vivo* co-cultured MDSCs with T cells were assessed for proliferation and apoptosis of T cells and differentiation to Tregs. Plasma factors and mRNA levels were analyzed by cytokine-bead assay and qRT-PCR.

**Results:**

Frequencies of MDSCs and Tregs were significantly increased (*p* = 0.011 and *p* = 0.02) with decreased CD4 T cells (*p* = 0.01) in sepsis than w/o sepsis and healthy controls (HCs) (*p* = 0.000, *p* = 0.07, and *p* = 0.01) at day 0 and day 7. In sepsis patients, MDSCs had increased IL-10, Arg1, and iNOS mRNA levels (*p* = 0.016, *p* = 0.043, and *p* = 0.045). *Ex vivo* co-cultured MDSCs with T cells drove T-cell apoptosis (*p* = 0.03, *p* = 0.03) with decreased T-cell proliferation and enhanced FOXP3^+^ expression (*p* = 0.044 and *p* = 0.043) in sepsis compared to w/o sepsis at day 0. Moreover, blocking the MDSCs with inhibitors suppressed FOXP3 expression. GM-CSF treatment in sepsis patients significantly decreased MDSCs and FOXP3^+^ Tregs but increased CD4 T-cell functionality and improved survival.

**Conclusion:**

MDSCs have an immunosuppressive function by expanding FOXP3^+^ Tregs and inhibiting CD4^+^ T-cell proliferation in sepsis. GM-CSF treatment suppressed MDSCs, improved T-cell functionality, and reduced Tregs in circulation.

## Introduction

Sepsis ranges from any infection to septic shock, and cirrhosis has been recognized as an independent mortality risk factor in septic shock patients (1). The development of sepsis in cirrhosis patients considerably increases both short- and long-term mortality due to immunological changes and systemic hemodynamics, while in-hospital mortality of cirrhosis patients with septic shock is higher, i.e., more than 70% (2).

Liver cirrhosis generally shows cirrhosis-associated immune dysfunction (CAID) by altering both innate and adaptive immunity (3). Impaired neutrophil and monocyte phagocytic ability and decreased HLADR expression of monocytes are the hallmarks of alcoholic liver cirrhosis patients. This disability in innate immunity leads to dysfunctional B and T cells in cirrhosis patients (4).

It is observed that defective myelopoiesis drives immature myeloid cells in the generation of myeloid-derived suppressor cells (MDSCs), instead of monocytes, dendritic cells (DCs), and neutrophils (5). MDSCs are heterogeneous in nature, and based on the expression of CD14, CD15, CD11b, CD33, and HLADR, they are distinctly characterized as of monocytic (M-MDSCs; CD14^+ve^CD11b^hi^CD33^+ve^HLADR^lo^) and granulocytic (G-MDSCs; CD14^−ve^ CD15^+ve^CD11b^hi^CD33^+ve^HLADR^lo^) lineages (6). However, M-MDSCs and G-MDSCs drive their suppressive activities in two different ways. M-MDSCs secrete low ARG1 but suppress other cells by iNOS-mediated STAT1 nitration in an antigen-specific and non-specific manner (7). While G-MDSC function specifically in an antigen-specific manner with hyperactivation of ARG1, reactive oxygen species (ROS), NO, and superoxide producing peroxynitrite (PNT) (8). Furthermore, like Tregs, MDSCs are also suppressive in nature. In fact, both MDSCs and Tregs support each other: Tregs control the differentiation and function of MDSCs, while in return MDSCs help in Treg expansion (9, 10).

However, in the tumor microenvironment, secreted granulocyte-macrophage colony-stimulating factor (GM-CSF) recruits PD-L1 expressing immune-suppressive MDSCs, and blocking of GM-CSF reduced the IDO and PD-L1 expression in liver MDSCs (11). However, it was reported earlier that adding GM-CSF to the standard care reduced the infectious complications and shortened the antibiotic therapy duration in abdominal sepsis patients (12, 13). GM-CSF not only improves total leukocyte counts (TLCs) but also reversed the monocytic deactivation by increasing HLADR and TLR4 expression in sepsis patients. Furthermore, GM-CSF treatment was also correlated with increased anti-inflammatory cytokine production with less need for mechanical ventilation and longer hospital stay (12).

However, there is limited knowledge about MDSCs and their functionality in liver cirrhosis patients with and w/o sepsis. Therefore, the aim of the present study was i) to investigate the role of MDSCs in immune dysfunction and ii) modulation of MDSCs, T cells, and Tregs with GM-CSF therapy in decompensated cirrhosis (DC) patients with sepsis, which may have an impact on disease pathogenesis and patient survival.

## Methods

### Study Groups and Blood Sampling

A total of 164 DC patients were enrolled: without (w/o) sepsis** **(n = 62), with sepsis (n = 72), with sepsis and treated with GM-CSF** **(n = 30), and healthy controls (HCs; n = 15) at the Institute of Liver and Biliary Sciences (ILBS), New Delhi, between 2017 and 2020 (**Supplementary Figure 1**). In an ongoing randomized controlled trial, DC patients with sepsis were given 250 μg of GM-CSF intravenously for about 6 h daily for 5 days. All the patients received standard medical treatment that included nutrition, antibiotics, and supportive care as part of standard medical treatment.

This study was approved by the Research and Institutional ethics committee with IEC No. IEC/2016/45/NA/C2, and informed consent was obtained from all the subjects enrolled in the study. In this longitudinal study, patients were closely monitored from admission and studied at baseline and on days 3 and 7. Patients with a history of any hepatitis infection [hepatitis B virus (HBV), hepatitis C virus (HCV), etc.], with HCC or any other site malignancy or any other comorbidities, and gave no consent were excluded from the study. This study was carried out in accordance with the ethical standards of the Declaration of Helsinki.

The clinical as well as biochemical assessment of all the enrolled patients with or w/o systemic inflammatory response syndrome (SIRS) or sepsis was done according to the treating physician’s direction. Patients were recruited based on the following criteria: DC w/o sepsis was diagnosed when there was no evidence of SIRS or infection on any of the cultures and ultrasonography; DC with sepsis was diagnosed based on the presence of SIRS and infection, confirmed either by cultures or by imaging.

Four criteria for the diagnosis of SIRS were used (14):

a) Temperature >38.0°C or <36.0°C

b) TLC <4 or >12 × 10^9^/L

c) Pulse > 90 beats/min

d) Respiratory rate (RR) >20 or <32 beats/min

### Blood Sampling

Peripheral blood measuring 15–20 ml was collected in ethylenediaminetetraacetic acid (EDTA)-coated tubes from patients and healthy subjects at the time of recruitment in the study. From part of the blood, plasma was separated and stored at −80°C for assessment of cytokines, endotoxin, and other circulating analytes.

### Multiparametric Whole Blood Immune Phenotyping

MDSCs, T cells, and Tregs were characterized in whole blood using specific antibodies against surface and intracellular markers labeled with different fluorochromes. MDSCs were characterized using anti-CD14 at 1:40 dilution (Clone M5E2, fluorescein isothiocyanate (FITC), 555397; BD Biosciences, San Jose, CA, USA), anti-CD11b at 1:100 dilution (Clone ICRF44, antigen-presenting cells (APCs), 301322; BioLegend, San Diego, CA, USA), anti-CD33 at 1:40 dilution (Clone P67.6, PE-Cy7, BioLegend 366618), and anti-HLADR at 1:40 dilution (Clone L243, BV510, BioLegend 307646); naïve and memory T cells by anti-CD4 at 1:40 dilution (Clone SK3, PE-Cy7 BD Biosciences 557852), anti-CD8 at 1:40 dilution (Clone SK1,V510, BD BioLegend 344732), anti-CCR7 at 1:20 dilution (Clone G043H7, FITC, BioLegend 353216), and anti-CD45RA at 1:20 dilution (Clone HI100, V410, BioLegend 304130); and Tregs by anti-CD4 at 1:40 dilution (Clone OKT4,V410, BioLegend 317434), anti-CD25 at 1:20 dilution (Clone, PE-Cy5, BioLegend 302608), anti-FOXP3 (Clone 259D/C7, PE, BD Biosciences 560046), and anti-CD127 (Clone 259D/C7, APC, BioLegend 351316). **Supplementary Table 1** shows the markers for types of T cells, MDSCs, and Tregs.

Briefly, 100–150 µl of whole blood was incubated with appropriate concentrations of surface antibodies for 30 min at room temperature (RT) in the dark. After that, cells were fixed with 200 µl of Cytofix (BD Biosciences, #554714) for 10 min and washed twice with 1× perm wash. Furthermore, cells were incubated with intracellular antibodies for 30 more minutes at RT and washed with 1× phosphate-buffered saline (PBS). A minimum of 100,000 events were acquired using a BD FACS VERSE, and all relevant data were analyzed by using FlowJo software version 10.

### Analysis of Plasma Analytes Using Cytokine Multiplex Bead Array Assay

To understand the significance of various cytokines and growth factors linked to sepsis as well as MDSC, we investigated the concentrations of forty-one plasma cytokines, chemokine and growth factors such as IL-1RA, IL-1β, IL-2, IL-12 (p40), IL-27, IL-18, IL-10, IL-17A, IFN-γ, TNF-α, IL-4, IL-6, IL-33, TGF-β1, IL-8, MIF, MIP-1α, MIP-1β, MIP-3α, ITAC, MCP-1, FRACTALKINE, ENA78, IP-10, EOTAXIN, Angiopoietin, MCSF, G-CSF/CSF3, GM-CSF, HGF, LEPTIN, TPO, VEGF-A, MMP7, MMP8, MMP1, MMP12, MMP13, P SELECTIN, E SELECTIN, and TREM1 in different patient groups by using multiplex PROCATAPLEX (Thermo Fisher, Waltham, USA), following the complete details on Luminex xponent 3.1TM Rev. 2 (Luminex Corporation, Austin, TX, USA). A standard curve was drawn using the standards provided in the kit. Values in samples were determined corresponding to the standard curve drawn. The lower detection limit of each cytokine is listed in **Supplementary Table 2**.

### Preparation of Myeloid-Derived Suppressor Cells, T Cells, and Adherent Monocytes

Part of the whole blood collected from patients of different groups was used for sorting MDSCs and CD4^+^ T cells. Cells were stained with anti-CD11b APC, anti-CD33 PE-Cy7, anti-HLADR V410, and anti-CD4 FITC antibodies and incubated for 30 min at RT in the dark, which is followed by red blood cell (RBC) lysis. Sorting of MDSC and T cells was done using BD FACS ARIA Fusion (BD Biosciences).

However, adherent monocytes were generated using 5 × 10^6^ isolated peripheral blood mononuclear cells (PBMCs). Cells were plated in complete Roswell Park Memorial Institute (RPMI) 1640 media with 10% fetal bovine serum (FBS) for 2 h. After 2 h, supernatant and non-adherent cells were removed, and adherent monocytes were removed for further experiments.

### Myeloid-Derived Suppressor Cell Functionality

#### T-Cell Apoptosis

To analyze the functional modulatory role of MDSCs, sorted CD4^+^ T cells were cultured with or without fluorescence-activated cell sorting (FACS)-sorted MDSCs or adherent monocytes for 3 days. For assessment of apoptosis of T-cell cultures, cells were removed after 3 days and stained with Annexin V for 15 min followed by propidium iodide (PI). The degree of apoptosis was assessed by flow cytometry using PI and Annexin V. Apoptotic cells were defined as PI−veAnnexin V+ve cells.

#### T-Cell Proliferation

The proliferation of T cells was assessed using a fluorescent cell staining dye, i.e., carboxyfluorescein succinimidyl ester (CFSE). A 96-well plate was coated overnight with anti-CD3 (0.5 µg/ml). Sorted CD4 T cells measuring 0.5 × 10^6^/ml were stained with 1 µl of CFSE (5 mM of stock solution, #21888; Sigma-Aldrich, St. Louis, MO, USA) for 15 min in the dark, cells were centrifuged at 1,200 rpm for 7 min, and the supernatant was discarded. CFSE labeling was stopped by the addition of RPMI 1640 medium (GIBCO, Thermofisher Scientific, Massachusetts, United States #22400-089) supplemented with 10% FBS (Gibco). After several washes, CFSE-labelled T cells along with soluble anti-CD28 (1 μg/ml) were plated alone, with sorted MDSCs and adherent monocytes in anti-CD3 coated wells, which were later incubated for 3–5 more days. The percentage of proliferation was assessed by flow cytometry using CFSE.

### Generation of Tregs Under Th0 and Th17 Conditions

For the Th0 condition, FACS-sorted CD4 T cells were cultured alone or with MDSCs (1:1) for 3 days under the Th0 condition [anti-CD3 (1 μg/ml) and anti-CD28 (1 μg/ml)]. After 3 days, Tregs (CD4^+^FOXP3^+^ cells) were determined using flow cytometry.

For the Th17 condition, FACS-sorted CD4 T cells were cultured under Th17-proliferating conditions, i.e., recombinant TGF-β (5 ng/ml, #sc-52893; Santa Cruz Biotechnology, Inc., Dallas, TX, USA), and IL-6 (20 ng/ml, #200-06; PeproTech, Cranbury, NJ, USA) along with anti-CD3 (1 μg/ml) and anti-CD28 (1 μg/ml) for 3 days. After 3 days, these cells were incubated alone or with MDSC (1:1 ratio) for 3 more days in RPMI 1640 complete media. After that, cells were stained with anti-IL-17 PE-Cy7 and anti-FOXP3 PE using intracellular cytometric analysis and determined using flow cytometry.

### Inhibition of Myeloid-Derived Suppressor Cells *via* Inhibitors

To unravel the mechanism of the Treg expanding effect of MDSCs, CD4 T cells were cultured with MDSCs (at a 1:1 ratio) under the Th0 condition for 72 h in the presence or absence of recombinant TGF-β (10 μg/ml, #sc-52893; Santa Cruz Biotechnology, Inc.), l-NMMA (500 μM, Sigma-Aldrich, #M7033), an iNOS inhibitor, and nor-NOHA (500 μM, Sigma-Aldrich, #399275), an Arg1 inhibitor. After that, cells were stained with anti-FOXP3 PE using intracellular cytometric analysis.

### Quantitative RT-PCR Analysis

FACS-sorted MDSCs and adherent monocytes were used to isolate total RNA using a miRVANA kit (Thermo Fisher, #AM1560). cDNA was prepared using a High-Capacity cDNA Kit (ABI, Thermo Fisher Scientific, Waltham, MA, USA; #4368813). The expression of various genes like IL-10, iNOS, and Arginase1 was checked. The primer sequence genes are listed in **Supplementary Table 3**. 18S RNA served as an endogenous control for normalization. Relative expression was analyzed using SYBR Green (Thermo Fisher, # A25742) in a VIIA7 real-time PCR machine (ABI, Whitefield, India).

### Statistical Analysis

Data were analyzed using the statistical software Prism (version 6; GraphPad Software, San Diego, CA, USA) and SPSS version 22 (IBM Corp, Ltd., Armonk, NY, USA). The comparison for continuous data is carried out by using the one-way ANOVA/Kruskal–Wallis test followed by probability adjustment by the Mann–Whitney test or by Bonferroni test *post-hoc* comparison as appropriate, and it is represented as mean ± SD. *p*-values <0.05 were considered statistically significant. Data with unequal distribution were used as medians. Moreover, this multinomial logistic regression was also applied along with diagnostic tests (receiver operating characteristic (ROC) curve).

## Results

Baseline characteristics of 164 DC patients, 62 patients w/o sepsis (age 48 ± 5 years, 87% male), 72 patients with sepsis (42 ± 9, 97% male), and 15 age-matched healthy controls were analyzed at the time of admission and enrolment in the study. Alcohol was the predominant etiology (70%) in DC patients. Sepsis patients showed a significant increase in total bilirubin, aspartate aminotransferase (AST) levels, international normalized ratio (INR), procalcitonin (PCT), lactate, MELD Na, and creatinine as compared to w/o sepsis patients in **Table 1**. The whole blood immune scan revealed lymphopenia but increased neutrophils in sepsis patients (**Supplementary Figures 2A, B**).

**Table 1:** Baseline clinical as well as biochemical characteristics of study groups.

Median and range	Healthy control (N = 15)	DC w/o sepsis (N = 62)	DC with sepsis (N = 72)	*p*-Value in betweenw/o and with sepsis
Age	32 (20–40)	48 (22–62)	44 (29–60)	0.07
Male:female	11:4	54:8	70:2	–
Total bilirubin (mg/dl)	1 (0.3–1.5)	4.5 (1.6–24)	14.5 (2.2–31.7)	0.00
AST (IU/ml)	20 (5–40)	59.75 (31–510)	114 (31–1037)	0.05
ALT (IU/ml)	25 (10–40)	34.5 (20–634)	41.5 (11–233)	1.00
INR (s)	1 (0.8–1.2)	1.66 (1.1–3.3)	2.58 (1.58–6.75)	0.00
PCT (ng/ml)	0.8 (0.2–2)	0.42 (0.04–3.25)	8.4 (0.07–88.4)	0.03
Lactate (mmol/L)	1.5 (1–2)	1.4 (0.6–5.2)	2.1 (0.2–13.3)	0.02
Sodium (mmol/L)	140 (136–145)	133 (124.3–142.7)	131 (113.2–148.4)	1.00
Creatinine (mg/ml)	0.6 (0.2–1)	0.86 (0.3–2.9)	1.3 (0.3–5.18)	0.04
MELD Na	8 (6–10)	23 (10–37)	32.5 (14–40)	0.00
SIRS criteria				
TLC (10^9^ L)	6 (4–11)	6.3 (3.1–19.8)	12.65 (2.7–43.6)	0.00
Pulse (/min)	70 (60–100)	84 (60–110)	94 (62–132)	0.00
RR (/min)	14 (12–16)	20 (16–24)	22 (14–34)	0.02
Temperature (F)	98 (97–99)	98.2 (97–98.9)	98.4 (96–100)	1.00
Differential leukocyte count				
Neutrophils (%)	60 (40–75)	70 (59–89)	81 (36–95)	0.00
Lymphocytes (%)	30 (20–45)	16 (3.4–36)	8 (1–29)	0.00
Monocytes (%)	5 (2–10)	11 (2–18)	8 (2–30)	0.03

DC, decompensated cirrhosis; AST, aspartate aminotransferase; ALT, alanine aminotransferase; INR, international normalized ratio; PCT, procalcitonin; MELD Na, model for end-stage liver diseases-sodium; SIRS, systemic inflammatory response syndrome; TLC, total leukocyte count; RR, respiratory rate.

### Increase in Myeloid-Derived Suppressor Cells in Sepsis Patients

#### At the Time of Admission (Day 0)

**Figure 1:**
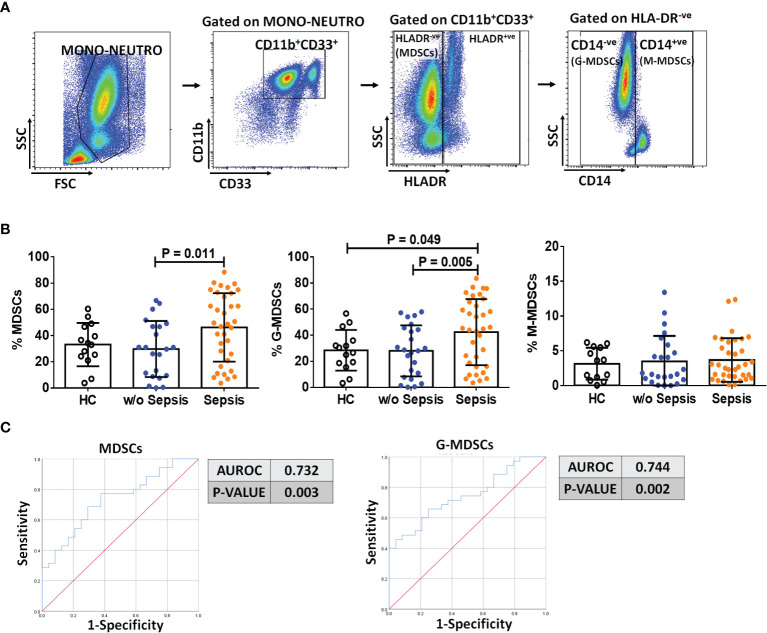
Identification of MDSCs in patient groups. **(A)** Sequential gating strategy for identification of MDSCs and their subsets using flow cytometry. MDSCs are characterized as CD11b^+^CD33^+^HLADR^−ve^. **(B)** Scatter dot plot shows %frequency of MDSCs, G-MDSCs, and M-MDSCs. **(C)** ROC curve shows high specificity and sensitivity of MDSCs and G-MDSCs in sepsis patients compared to w/o sepsis patients. Results are expressed as the mean ± SD. **(B)** One-way ANOVA/Kruskal–Wallis test followed by probability adjustment by the Mann–Whitney. **(C)** ROC curve *via* SPSS. MDSCs, myeloid-derived suppressor cells; ROC, receiver operating characteristic.

Based on the gating strategy, expression of MDSCs (CD11b^+^CD33^+^HLADR^−ve^) was significantly increased (*p* = 0.011) in sepsis patients compared to w/o sepsis patients. We have further characterized subsets of MDSCs, i.e., G-MDSCs and M-MDSCs. Expression of G-MDSCs (CD11b^+^CD33^+^HLADR^−ve^CD14^−ve^) was found to be significantly increased in sepsis patients (*p* = 0.005) compared to w/o sepsis patients, while M-MDSCs (CD11b^+^CD33^+^HLADR^−ve^CD14^+ve^) showed no significant difference between the groups (**Figures 1A, B**). A logistic regression model positively predicted an increase in MDSCs and G-MDSCs with high sensitivity and specificity (0.732, *p* = 0.003 and 0.744, *p* = 0.002) in sepsis patients compared to w/o sepsis patients (**Figure 1C**). A comparison of gating strategies is demonstrated by (6, 15) (**Supplementary Figure 3A**).

#### At Follow-Up Time Points

On follow-up on day 3 and day 7, there was a significant decrease in MDSCs and G-MDSCs in sepsis patients on day 7 (*p* = 0.04 and *p* = 0.01) compared to day 0, but no difference in M-MDSCs (**Supplementary Figure 3B**). There was no change on day 3.

### Decrease in CD4^+^ T Cells and Its Subsets While Increase in Tregs in Sepsis Patients

#### At the Time of Admission (Day 0)

**Figure 2:**
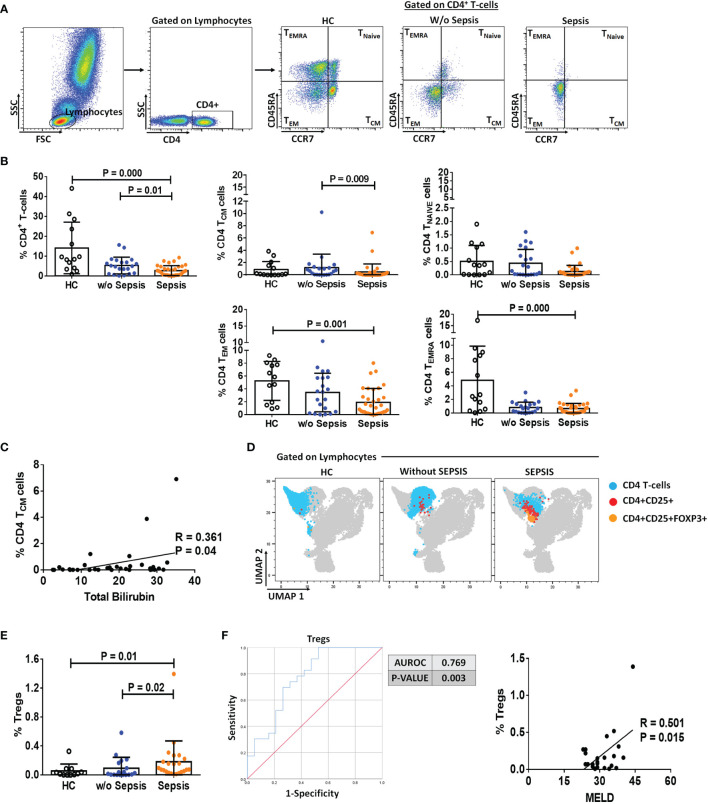
Identification of CD4 T cells, their subsets, and Tregs in patient groups. **(A)** Sequential gating strategy for identification of CD4 and its subsets using CCR7 and CD45RA, i.e., T_CM_, T_NAIVE_, T_EM,_ and T_EMRA_. Scatter dot plot shows %frequency of CD4 T cells in patient groups. **(B)** Scatter dot plot shows %frequency of T_CM_, T_NAIVE_, T_EM_, and T_EMRA_. **(C)** Correlation between %CD4 T_CM_ and total bilirubin in sepsis group. **(D)** UMAP visualization of pooled lymphocytes of HCs and patients w/o and with sepsis for characterization of Tregs. **(E)** Scatter dot plots show %frequency of Tregs. **(F)** ROC curve shows high specificity and sensitivity of Tregs in sepsis group compared to w/o sepsis group. Also, correlation of %Tregs with MELD in sepsis group. Results are expressed as the mean ± SD. **(A, B, E)** One-way ANOVA/Kruskal–Wallis test followed by probability adjustment by the Mann–Whitney. **(C–F)** Scatter diagram showing the regression line. UMAP, Uniform Manifold Approximation and Projection; HCs, healthy controls; ROC, receiver operating characteristic; MELD, model for end-stage liver diseases.

The presence of MDSCs modulates T-cell differentiation (10); therefore, to analyze the impact of MDSCs on CD4 T cells and T-cell differentiation, we have used CD45RA and CCR7 markers to evaluate the presence of naïve, T_CM_, T_EM_, and T_EMRA_ in circulation (**Figure 2A**). Sepsis patients showed a significant decrease in the total percentage of CD4**^+^** T cells as compared to w/o sepsis patients and HCs (*p* = 0.000 and *p* = 0.01). When CD4^+^ T-cell subsets were analyzed, it was observed that although naïve T cells were not found significantly different between the groups, T_CM_ was decreased in sepsis patients compared to w/o sepsis patients (*p* = 0.009) (**Figure 2B**). In fact, T_EM_ and T_EMRA_ populations were also decreased in the sepsis group, but this difference was observed as compared to HCs only (*p* = 0.000 and *p* = 0.001) but not with w/o sepsis patients (**Figure 2B**). A decrease in T_CM_ in sepsis was also positively correlated with increased total bilirubin levels (*p* = 0.04) (**Figure 2C**).

Furthermore, Tregs were increased in sepsis patients (*p* = 0.02 and *p* = 0.01) compared to w/o sepsis patients and HCs (**Figures 2D, E**). A logistic regression model positively predicted Tregs with high sensitivity and specificity (0.769, *p* = 0.003) in both sepsis and w/o sepsis patients. However, Tregs were found positively correlated with MELD scores in sepsis patients (*p* = 0.015) (**Figure 2F**).

#### At Follow-Up Time Points

No difference in percentages of total CD4 T cells, T_NAIVE_ T_CM_, T_EMRA_, and T_EM_ was observed in follow-up between the groups. However, somehow percentage frequencies of Tregs were significantly decreased in sepsis patients on day 3 and day 7 (*p* = 0.002 and *p* = 0.008) compared to day 0, which could be the effect of normal medical treatment in sepsis patients (**Supplementary Figures 4A–D**).

### Myeloid-Derived Suppressor Cells Express More IL-10, Arg1, and iNOS

**Figure 3:**
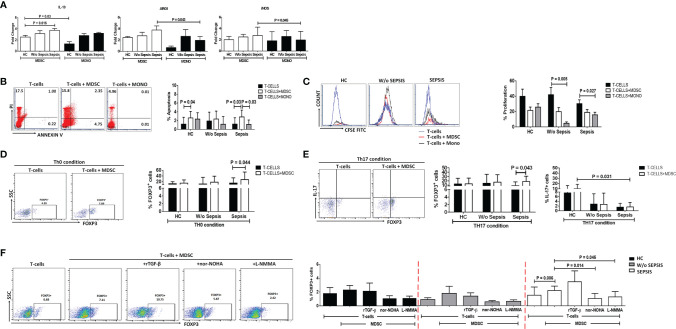
MDSCs action on T-cell functionality and Tregs. **(A)** Fold-change expression of IL-10, Arg1, and iNOS in sorted MDSCs (white color) and monocytes (black color) in the patient groups through qRT-PCR. **(B)** %Apoptosis using PI−veAnnexin V+ve and **(C)** %Proliferation using CFSE through flow cytometry in T cells cultured alone (black color) and with MDSCs (white color) and monocytes (gray color) in the patient groups. Expression of %FOXP3^+^ on CD4^+^ T cells **(D)** under Th0 condition and **(E)** under Th17-proliferating condition was observed in the patient groups when T cells were cultured alone (black color) and with MDSCs (white color). **(F)** Expression of %FOXP3^+^ on CD4^+^ T cells in T cells cultured with MDSCs along with stimulations, i.e., rTGF-β, l-NMMA, and nor-NOHA in the patient groups. HCs (black color), without sepsis (gray color), and with sepsis (white color). Results are expressed as the mean ± SD. **(A)** One-way ANOVA/Kruskal–Wallis test followed by probability adjustment by the Mann–Whitney. **(B–E)** Mann–Whitney/t-test within the patient groups. **(F)** Kruskal–Wallis test within the group along with the multiple comparisons. MDSC, myeloid-derived suppressor cell; CFSE, carboxyfluorescein succinimidyl ester.

Sorted MDSCs from sepsis patients showed increased IL-10, ARG1, and iNOS expression; however, a significant increase was observed in IL-10 compared to that in HCs (*p* = 0.016). As MDSCs have monocytic and granulocytic lineages and M-MDSCs at present, which are known to play an immunosuppressive role, i.e., suppression of T cell, Treg expansion, and disease severity (9), we have compared monocytes (not neutrophils) with MDSCs. When IL-10, ARG1, and iNOS expression in MDSCs was compared with that in monocytes, fold-change expression of ARG1 and iNOS was found significantly increased in sepsis MDSCs (*p* = 0.043 and *p* = 0.045) compared to sepsis monocytes but has no difference in IL-10 expression (**Figure 3A**). However, plasma levels of IL-10, IL-6, and IL-8 were significantly increased in sepsis compared to HCs and w/o sepsis patients. We have also analyzed MDSC-associated plasma factors including VEGF-A, IP-10, IL-6, IL-8, IL-10, TGF-β1, MIP-3α, IL-4, IL-27, IL-1β, IL-17A, TNF-α, and GM-CSF. We observed that MIP-3α and IL-1β were decreased in sepsis, MIP-3α was negatively correlated with MDSCs/G-MDSCs, and IL-1β and IP-10 were positively correlated with M-MDSCs in sepsis patients (**Supplementary Figures 5A–C**).

### Myeloid-Derived Suppressor Cells Suppressed T-Cell Functionality

To check the suppressive effect of MDSCs on T cells, FACS-sorted MDSCs and CD4 T cells were *ex vivo* co-cultured and analyzed for CD4 T-cell apoptosis and proliferation (**Supplementary Figure 6**). To know whether MDSCs and monocytes have a similar effect on T-cell apoptosis and proliferation, we have additionally co-cultured T cells with monocytes.

#### At the Time of Admission (Day 0)

We have observed an increase in apoptosis of T cells and a decrease in T-cell proliferation in *ex vivo* cultured MDSCs+ T cells in sepsis patients compared to w/o sepsis patients and HCs (**Figures 3B, C** and **Supplementary Figure 7A**). But monocytes did not show suppressive ability when co-cultured with T-cells, suggesting MDSCs have immunosuppressive ability in sepsis but not monocytes..

But monocytes did not show suppressive ability when co-cultured with T-cells, suggesting MDSCs have immunosuppressive ability in sepsis but not monocytes. 

#### At Follow-Up Time Points

We found no difference in follow-up between the groups in T-cell apoptosis and proliferation (**Supplementary Figures 7B, C**).

### Myeloid-Derived Suppressor Cells Induces FOXP3^+^ Expression on T Cells

*Ex vivo* cultured T cells with MDSCs in the Th0 condition (without the presence of any T-cell stimulant) showed increased expression of CD4^+^FOXP3^+^ (*p* = 0.044) in sepsis patients, but no such increased expression was observed in HCs and w/o sepsis patients (**Figure 3D**).

Furthermore, it was observed that in the presence of Th17-proliferating conditions [in presence of recombinant TGF-β (5 ng/ml) and IL-6 (20 ng/ml)], MDSCs induce more CD4^+^FOXP3^+^ expression on T cells (*p* = 0.043) in sepsis patients, while IL-17 producing T cells were minimal in disease condition compared to that in HCs (*p* = 0.031) (**Figure 3E**). We concluded that MDSCs increase the Treg expression and suppress Th17 cells, causing Treg/Th17 imbalance in DC patients with sepsis.

#### At Follow-Up Time Points

T cells cultured with MDSCs in the Th0 condition showed no difference in expression of CD4^+^FOXP3^+^, but an expression of CD4^+^FOXP3^+^ cells in the Th17 condition significantly decreased on day 3 compared to day 0 in w/o sepsis patient (**Supplementary Figure 8A, B**).

### Blocking the Myeloid-Derived Suppressor Cells Suppresses the Expression of FOXP3^+^ Tregs

MDSCs increased FOXP3 expression on CD4^+^ T cells in sepsis patients (*p* = 0.006). As MDSCs suppress *via* Arginase1 and iNOS, we have further explored the role of MDSC blockers: l-NMMA (iNOS inhibitor) and nor-NOHA (Arg1 inhibitor) on CD4^+^FOXP3^+^ T cells. By blocking MDSC activity with nor-NOHA and l-NMMA, there was a significant decrease in CD4^+^FOXP3^+^ T cells (*p* = 0.014 and *p* = 0.045) in sepsis patients, but no such significant difference was observed in w/o sepsis patients and HCs (**Figure 3F**).

#### At Follow-Up Time Points

MDSC inhibitors show a significant decrease in the expression of CD4^+^FOXP3^+^ on day 3 (*p* = 0.03 and *p* = 0.03) in sepsis patients compared to day 0, but no difference was observed in w/o sepsis patients (**Supplementary Figure 8C**).

### Granulocyte-Macrophage Colony-Stimulating Factor Treatment Suppresses Myeloid-Derived Suppressor Cells and Tregs in Sepsis Patients

GM-CSF is known as a stimulant for the bone marrow to produce myeloid cells and helps in the proliferation of myeloid cells but also helps in their proliferation (11, 12). However, the effect of exogenous treatment of GM-CSF in the modulation of MDSCs and Tregs was not explored in sepsis patients. We have analyzed 30 sepsis patients who were given 250 μg of GM-CSF intravenously over 6 h for 5 days along with the standard care. Blood samples were collected post 12 h after GM-CSF administration (day 1). Baseline as well as follow-up clinical and biochemical characteristics of sepsis patients with and without GM-CSF treatment were analyzed (**Supplementary Table 4**).

**Figure 4:**
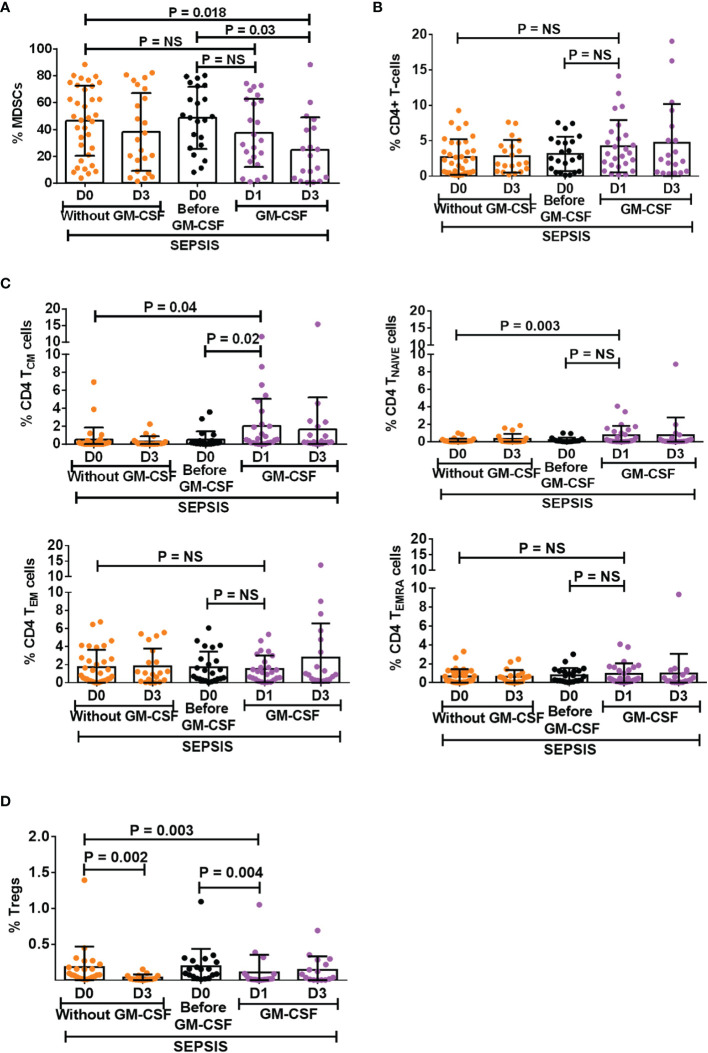
Effect of GM-CSF treatment on the expression of MDSCs, CD4 T cells, its subsets, and Tregs. Scatter dot plot shows an expression of **(A)** %MDSCs; **(B)** %CD4 T cells; **(C)** CD4 T-cell subsets, i.e., T_CM_, T_NAIVE_, T_EM_, and T_EMRA_; and **(D)** %Tregs in sepsis patients without GM-CSF (D0 and D3), before GM-CSF (black color), and with GM-CSF (D1 and D3). Results are expressed as the mean ± SD. **(A–D)** One-way ANOVA/Kruskal–Wallis test followed by probability adjustment by the Mann–Whitney *via* SPSS. GM-CSF, granulocyte-macrophage colony-stimulating factor; MDSCs, myeloid-derived suppressor cells.

After day 1 of GM-CSF therapy, neutrophils were decreased with no change in monocyte numbers but with an increase in HLADR expression on monocytes (**Supplementary Figure 9A**). Furthermore, after GM-CSF therapy on days 1 and 3, MDSCs were decreased in sepsis patients. (**Figure 4A**), although the effect was more on G-MDSCs and not on M-MDSCs (**Supplementary Figure 9B**). GM-CSF therapy also showed its impact on CD4^+^ T cells and their subsets. We found an increase in CD4 expression on day 1 after GM-CSF therapy, while both T_NAIVE_ and T_CM_ were found to be significantly increased in the GM-CSF group (*p* = 0.04 and *p* = 0.003) compared to the group without GM-CSF (**Figures 4B, C**). Furthermore, the percentage frequency of Tregs was significantly decreased in the GM-CSF group (*p* = 0.003) compared to without GM-CSF (**Figure 4D**).

### Granulocyte-Macrophage Colony-Stimulating Factor Treatment Reverses the Effect of Myeloid-Derived Suppressor Cells on T Cells and Tregs

**Figure 5:**
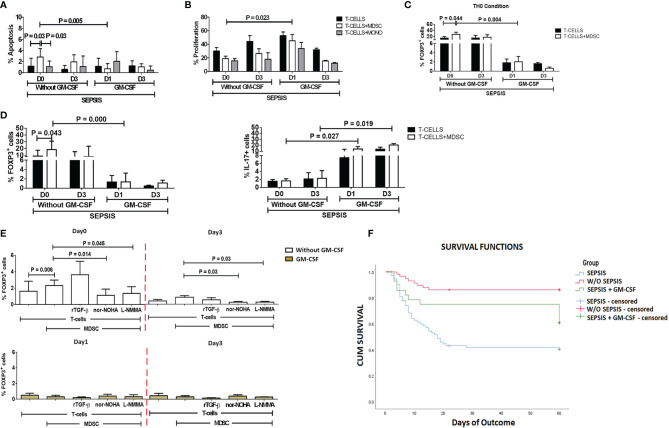
Effect of GM-CSF treatment on T-cell functionality and Treg expression. Bar diagrams show **(A)** %Apoptosis using PI−veAnnexin V+ve and **(B)** %Proliferation using CFSE in sepsis patients with and without GM-CSF in T cells cultured alone (black color), with MDSCs (white color), and with monocytes (gray color). Bar diagrams show expression of %FOXP3^+^ on CD4^+^ T cells **(C)** under Th0 condition and **(D)** under Th17-proliferating condition in T cells cultured alone (black color) and with MDSCs (white color). **(E)** Expression of %FOXP3^+^ on CD4^+^ T cells in T cells cultured with MDSCs along with stimulations with rTGF-β, l-NMMA, and nor-NOHA; %FOXP3+ Treg expression was observed in the groups with (light brown) and without GM-CSF (white color) at different time points. Results are expressed as the mean ± SD. **(A–D)** Mann–Whitney/t-test within the patient groups along with one-way ANOVA/Kruskal–Wallis test between the groups. **(E)** Kruskal–Wallis test within the group along with the multiple comparisons. **(F)** Survival in patient groups was observed using the Kaplan–Meier survival curves *via* SPSS. GM-CSF, granulocyte-macrophage colony-stimulating factor; CFSE, carboxyfluorescein succinimidyl ester; MDSCs, myeloid-derived suppressor cells.

After day 1 of GM-CSF therapy, *ex vivo* co-cultured MDSCs and T cells showed significantly decreased apoptosis of T cells (*p* = 0.005) as compared to those without GM-CSF therapy. Similarly, T-cell proliferation was significantly increased on day 1 in the GM-CSF group (*p* = 0.023) compared to the group without GM-CSF (**Figure 5B**). However, this effect was not observed on day 3 of GM-CSF therapy (**Figure 5A**).

Furthermore, expression of CD4^+^FOXP3^+^ on T cells (*p* = 0.004) in the Th0 condition was significantly decreased after GM-CSF day 1 therapy compared to without GM-CSF. At follow-up on day 3, CD4^+^FOXP3^+^-expressing T cells were constantly found to decrease in the GM-CSF group compared to the group without GM-CSF (**Figure 5C**).

Furthermore, in IL-17-proliferating conditions, MDSCs did not show an ability to induce the expression of CD4^+^FOXP3^+^ on T cells in the GM-CSF group (*p* = 0.000) compared to the group without GM-CSF. Similarly, till day 3, MDSCs were unable to induce CD4^+^FOXP3^+^ expression in the GM-CSF group compared to the group without GM-CSF. However, the percentage frequency of IL-17-expressing T cells was significantly increased in the GM-CSF group (*p* = 0.027) compared to the group without GM-CSF on day 1 and day 3 (**Figure 5D**).

### Granulocyte-Macrophage Colony-Stimulating Factor Treatment Reversed Myeloid-Derived Suppressor Cell Expression on Tregs

GM-CSF therapy in sepsis patients leads to a significant decrease in CD4^+^FOXP3^+^ Tregs. In *in vitro* co-cultured assay of CD4 T cells and MDSCs with l-NMMA (iNOS inhibitor) and nor-NOHA (Arg1 inhibitor) inhibitors, a decrease in CD4^+^FOXP3^+^ Tregs was observed after GM-CSF treatment on day 1 and day 3 (**Figure 5E**).

### Granulocyte-Macrophage Colony-Stimulating Factor Treatment Improves Survival of Sepsis Patients

Survival in DC patients with sepsis is mostly compromised. The Kaplan–Meier survival curves evidently proved that GM-CSF therapy along with standard care improved survival in sepsis patients compared to patients with only standard care (**Figure 5F**).

## Discussion

Our study observed an increase in MDSCs and Tregs in cirrhosis patients with sepsis, with a decrease in CD4 T cells. *Ex vivo* co-cultured MDSCs and T-cell experiments confirmed and supported the notion that MDSCs suppress T-cell functionality but expand FOXP3^+^ Tregs in sepsis patients.

**Figure 6:**
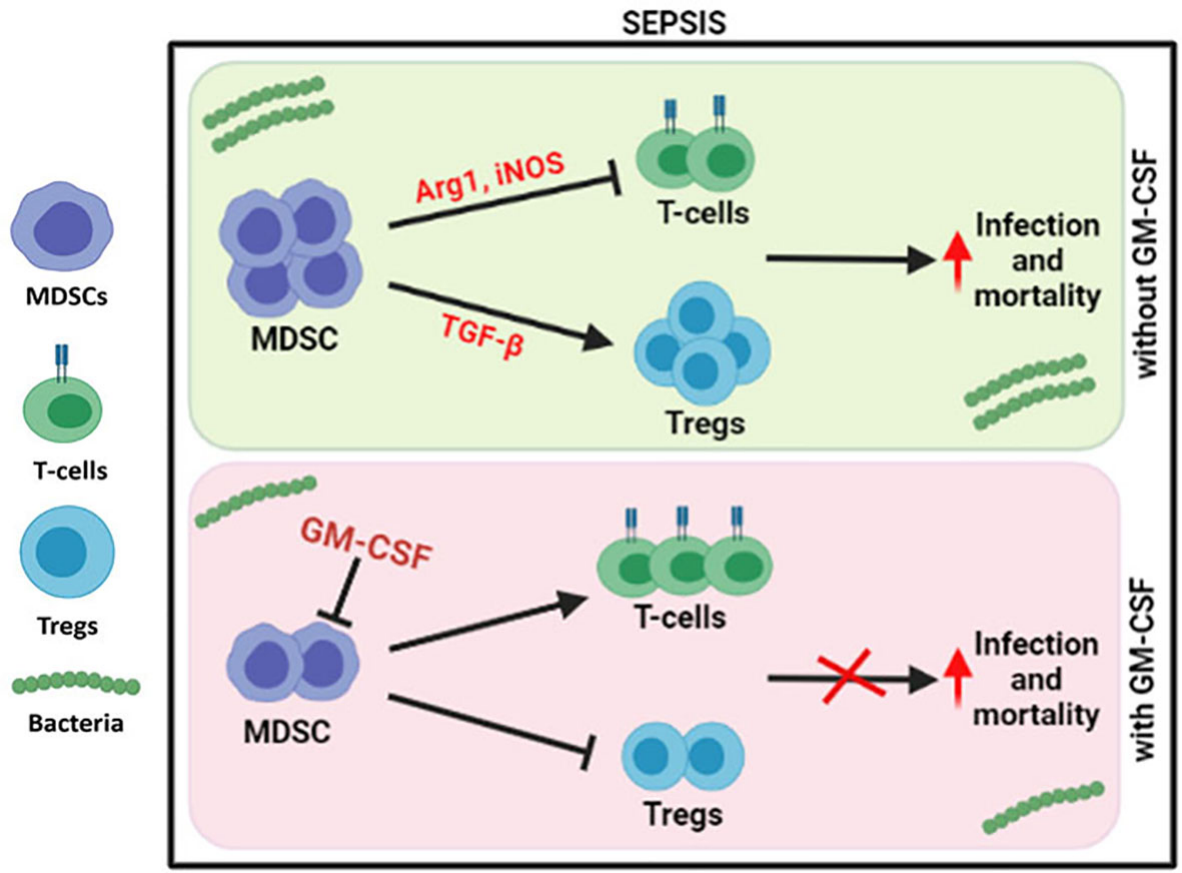
Modulation of immune cells in sepsis with GM-CSF. In DC sepsis patients, MDSCs suppresses the T-cells using Arg1 and iNOS but expands Tregs using TGF-b. With GM-CSF treatment, immune paralysis is reversed with decline in MDSCs, and Tregs with proliferation of T-cells.

Our group has earlier shown that G-CSF reduces the disease severity, delays the mortality of severe alcoholic hepatitis (SAH) patients (16), and mobilizes bone marrow-derived CD34^+^ cells in acute-on-chronic liver failure (ACLF) patients for hepatic regeneration (17). However, the focused role of GM-CSF was not addressed earlier, but in this study, usage of GM-CSF reversed the immune paralysis by suppressing the MDSCs and Tregs in sepsis patients (**Figure 6**).

Sepsis is described as organ dysfunction due to bacterial infections and induced by dysregulated host immune response resulting in a longer stay in hospitals, affecting the mortality rate (18). Immune dysfunction in liver cirrhosis patients is common, which enhances bacterial translocation from the gut to liver, resulting in endotoxemia, systemic inflammation, and septic shock (19). DC patients show leukopenia, which affects both T helper (Th) and cytotoxic T cells (Tc), monocytosis with altered function, neutrophils with impaired phagocytosis, B cells with memory B-cell dysfunction, and defective NK cells with reduced response to cytokine stimulation (4). Our study shows a decrease in both CD4 and CD8 T cells and monocytes but an increase in circulating neutrophils, MDSCs, and Tregs in DC patients with sepsis.

MDSCs are a heterogeneous population of cells, and their origin is either monocytic or granulocytic (6). Elevated levels of MDSCs have been positively correlated with severe sepsis or septic shock and longer stay of patients in the intensive care unit (ICU) (20), as MDSCs are known to have immunosuppressive activity *via* Arginase-1, iNOS, or ROS for inhibiting the functionality of immune cells, especially T cells. An increase in MDSCs acts as a potent inhibitor of T cell-mediated immunity in autoimmune hepatitis and cancer, which is attributed to the production of Arginase1, ROS, iNOS, and IL-10 (21, 22). An increase in hepatic CD11b^+^CD33^+^ MDSCs positively correlated with liver fibrosis and also linearly correlated with the tumor volume (23). Furthermore, peripheral blood MDSCs were also increased in cirrhosis and HCC patients (24). Furthermore, M-MDSCs were strongly correlated with raised ALT, AST, and decreased T-cell proliferation (25). It was earlier reported that M-MDSCs suppressed T-cell functions and antimicrobial-specific innate immune responses in ACLF patients (26). An increase in peripheral and intrahepatic G-MDSC populations was reported in ALC (Alcoholic liver cirrhosis) patients (27). In our study, we also found a significant increase in MDSCs, especially G-MDSCs, in cirrhosis patients with sepsis. *In vitro* co-cultured MDSCs with CD4 T cells suppressed the proliferation and enhanced apoptosis of CD4 T cells in DC with sepsis patients.

It is known that in the tumor microenvironment, many known circulating factors including IL-6, IFN-γ, TGF-β, VEGF, G-CSF, GM-CSF, M-CSF, and SCF induce the recruitment, accumulation, and activation of MDSCs (28) and modulate MDSCs to produce more of NO and ROS (22). Our study revealed a positive correlation of new molecules IL-1β and IP-10 with M-MDSC but a negative correlation of MIP-3α with MDSC and G-MDSC. Reduced IL-1β receptor binding ability or IL-1β levels reduces the accumulation and suppressive activity of MDSC resulting in augmentation of antitumor immunity and delayed tumor growth (29). Furthermore, chemokine CXCL10/IP-10 significantly increased in a mouse model of septic shock, as they cause the activation of chemokine receptor CXCR3, an important regulator of lymphocyte trafficking and activation (30). MIP-3α (macrophage inflammatory protein-3)/CCL20 is generally expressed on several immune cells but with a stronger chemotactic effect and interaction with chemokine receptor CCR6 on lymphocytes. CCL20/CCR6 axis regulates the activation and suppression of immune cells (31).

Both MDSC and Tregs are known suppressor cells and help each other; i.e., Tregs regulate the differentiation and function of MDSC *via* TGF-β, while MDSC helps in the expansion of Tregs in a colitis mouse model (7). In the rheumatoid arthritis mouse model, MDSC-derived IL-10 helps in the generation of Tregs but attenuates inflammation. It was found that MDSCs regulate Th17/Treg cells and control inflammation (8). Tregs/Th17 axis plays an important role in various diseases, and Treg/Th17 imbalance was known to be used in the pathogenesis of HBV-related liver cirrhosis and ACLF (32, 33). We have also shown that in DC patients with sepsis, MDSCs significantly enhanced the expression of FOXP3 on CD4^+^ T cells and behaved as Tregs, while stimulation with l-NMMA (iNOS inhibitor) and nor-NOHA (inhibitor of Arginase1) significantly suppresses the expansion of Tregs in sepsis patients. It clearly concludes that suppression of the immunosuppressive activity of MDSCs will decrease the expansion of CD4^+^FOXP3^+^ cells in sepsis.

Although we had earlier observed survival benefits of G-CSF in SAH and ACLF patients (16, 17), the role of another CSF moiety, i.e., GM-CSF, was explored in sepsis patients in this study.

It was observed earlier that administration of GM-CSF in sepsis patients reversed the monocytic deactivation by increasing HLADR and TLR4-induced cytokine production, as well as decreased the time of mechanical ventilation and length of hospital (34). *In vitro* mouse model showed that the combination of GM-CSF signaling blockade and gemcitabine suppressed the MDSC phenotype and functionality (22). In another study, both GM-CSF and G-CSF prevented diabetes by reducing MDSCs and Treg cells (35). Our results clearly suggested the benefit of GM-CSF therapy in sepsis patients, as it decreases the MDSCs, FOXP3 expression on CD4^+^ T cells, and the percentage of apoptotic T cells and increases CD4 T-cell proliferation. However, post-GM-CSF therapy on day 3, we did not observe any significant change in apoptosis and proliferation of CD4 T cells as compared to day 1. In sepsis patients, it is difficult to do a liver biopsy; therefore, our study was limited to peripheral blood. Survival rates in cirrhosis patients with sepsis are mostly compromised; however, standard care along with GM-CSF therapy has increased survival benefits in sepsis patients. This could be due to decreased MDSCs and Tregs and increased T_CM_ population. Although we have shown earlier survival benefits of G-CSF in ACLF and SAH patients, this study also showed the survival benefits after administration of GM-CSF in sepsis patients. The role of GM-CSF independent of the MDSCs can be best answered *via* mouse models, which act as a limitation to our study.

In summary, we conclude that the enhanced expression of MDSCs in DC with sepsis was found to be responsible for suppressing CD4^+^ T cell functionality as well as expanding the CD4^+^FOXP3^+^ Treg activity. Administration of GM-CSF in sepsis patients reduced the numbers of MDSCs and Tregs and improved T-cell functionality, which are beneficial for the survival of the patients.

## Data Availability Statement

The original contributions presented in the study are included in the article/**Supplementary Material**. Further inquiries can be directed to the corresponding authors.

## Ethics Statement

The protocol was approved by the institutional review board and ethics committee (IEC No. IEC/2016/45/NA/C2). The patients/participants provided their written informed consent to participate in this study.

## Author Contributions

RS collected clinical samples, performed all experiments after inputs from NT, and did the initial analysis. RM, VR, and SS helped in the recruitment and characterization of patients in each group. MI helped in performing the experiments, and GK helped in the statistical analysis. An initial draft of the manuscript was written by RS. SS, RM, SB, NK, and GR have provided inputs in the manuscript. NT revised, corrected, and finalized the manuscript. All authors listed have made a substantial, direct, and intellectual contribution to the work and approved it for publication.

## Funding

The study was partially supported by research funds from the Department of Science and Technology (DST) (SB/EF/02/2016 dated March 23, 2017), Government of India.

## Conflict of Interest

The authors declare that the research was conducted in the absence of any commercial or financial relationships that could be construed as a potential conflict of interest.

## Publisher’s Note

All claims expressed in this article are solely those of the authors and do not necessarily represent those of their affiliated organizations, or those of the publisher, the editors and the reviewers. Any product that may be evaluated in this article, or claim that may be made by its manufacturer, is not guaranteed or endorsed by the publisher.

## Glossary

**Table:**

MDSCs	myeloid-derived suppressor cells
CAID	cirrhosisassociated immune dysfunction
DC	decompensated cirrhosis
ICU	intensive care unit
IMCs	immature myeloid cells
DCs	dendritic cells
G-MDSCs	granulocytic MDSCs
M-MDSCs	monocytic MDSCs
iNOS	inducible nitric oxide synthase
ROS	reactive oxygen species
PNT	peroxynitrite
TGF-b	transforming growth factor beta
IL	interleukin
GM-CSF	granulocytemacrophage colony-stimulating factor
G-CSF	granulocyte colony-stimulating factor
M-CSF	macrophage colony-stimulating factor
VEGF	vascular endothelial growth factor
SCF	stem cell factor
ALT	alanine aminotransferase
AST	aspartate aminotransferase
ACLF	acute-on-chronic liver failure
SAH	severe alcoholic hepatitis
FOXP3	Forkhead box P3
IDO	indoleamine 2,3-dioxygenase
PD-L1	programmed cell death ligand 1
HC	healthy control
RCT	randomized controlled trial
HCC	hepatocellular carcinoma
SIRS	systemic inflammatory response syndrome
TLC	total leukocyte count
RR	respiratory rate
INR	international normalized ratio
EDTA	ethylenediaminetetraacetic acid
PBMCs	peripheral blood mononuclear cells
PBS	phosphate-buffered saline
FBS	fetal bovine serum
RPMI	Roswell Park Memorial Institute
LPS	lipopolysaccharide
RT	room temperature
RBC	red blood cells
PI	propidium iodide
CFSE	carboxyfluorescein succinimidyl ester
FACS	fluorescence-activated cell sorting
L-NMMA	Nw-methyl-L-arginine acetate salt
nor-NOHA	Nw-hydroxy-nor-Larginine diacetate salt
SPSS	Statistical Package for the Social Sciences
DLC	differential leukocyte count
PCT	procalcitonin
MELD Na	model for end-stage liver diseases-sodium
CD4+CD45RA+CCR7+	naïve T cells
TCM	CD4+CCR7+CD45RA−central memory T cells
TEM	CD4+CD45RA−CCR7−effector memory
TEMRA	CD4+CD45RA+CCR7 terminally differentiated T cells
