# Supplementary material for: Granulocyte-Macrophage Colony-Stimulating Factor Modulates Myeloid-Derived Suppressor Cells and Treg Activity in Decompensated Cirrhotic Patients With Sepsis
Source: Front Immunol. 2022 Jun 3;13:828949. doi: 10.3389/fimmu.2022.828949 (PMC9205181; doi:10.3389/fimmu.2022.828949)
Supplement: Supplementary file 1 [file DataSheet_1.docx]

**GMCSF modulates Myeloid derived suppressor cells and Tregs activity in decompensated cirrhotic patients with sepsis.**

*Supplementary Information*

Rashi Sehgal^1,3^, Rakhi Maiwall^2^, Vijayraghavan Rajan^2^, Mojahidul Islam^1^, Sukriti Baweja^1^, Navkiran kaur^3^, Guresh Kumar^4^, Gayatri Ramakrishna^1^, Shiv K Sarin^2*^, Nirupama Trehanpati^1*^

**Corresponding Authors:**

Dr. Nirupama Trehanpati, PhD

Professor, Department of Molecular and Cellular Medicine

Institute of Liver and Biliary Sciences

D-1 Vasant Kunj, New Delhi 110070, India

[trehanpati@ilbs.in](mailto:trehanpati@ilbs.in), [trehanpati@gmail.com](mailto:trehanpati@gmail.com)

ORCID: [0000-0002-6109-0033](https://orcid.org/0000-0002-6109-0033)

Dr. Shiv K Sarin, MD, DM, DSc (Hony.)

Senior Professor, Department of Hepatology

Institute of Liver and Biliary Sciences

D-1 Vasant Kunj, New Delhi 110070, India

E-mails: [shivsarin@gmail.com](mailto:shivsarin@gmail.com), shivsarin@ilbs.in

Tel.: +91-11-46300000,

ORCID: 0000-0002-0544-5610

**SUPPLEMENTARY TABLES:**

**Supplementary Table 1:** Markers for cell types: T-cells, MDSCs and Tregs.


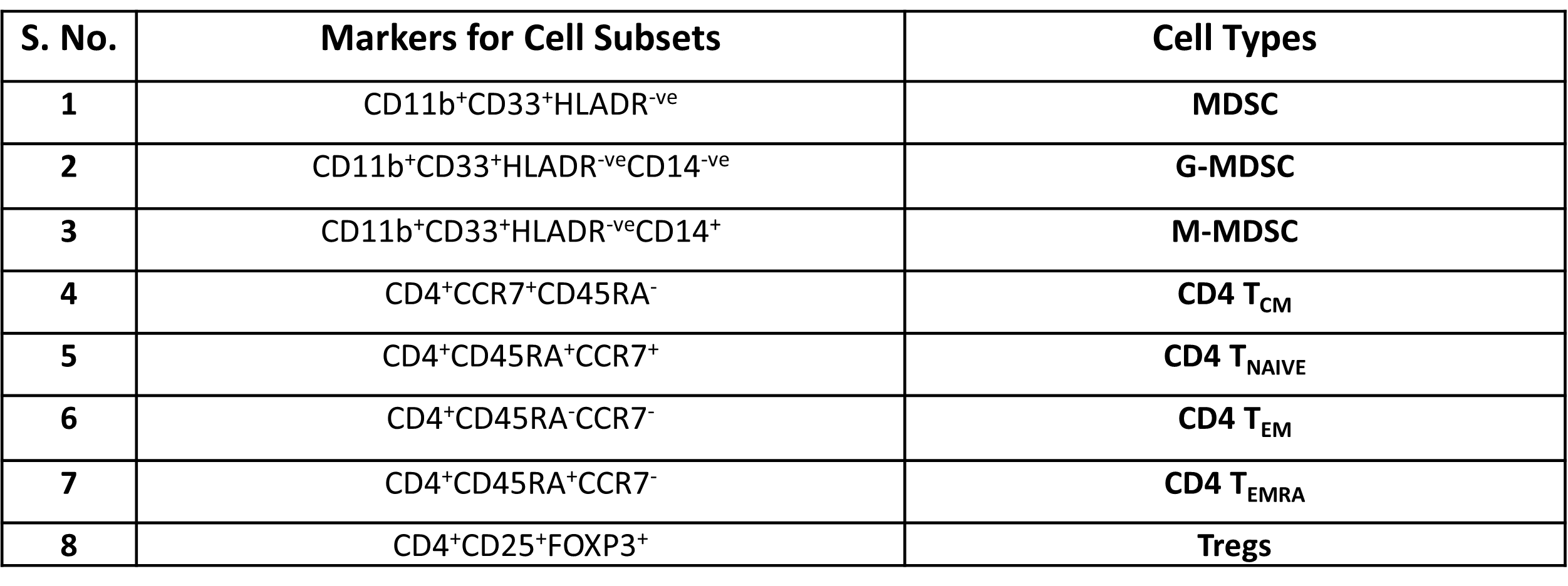


**Supplementary Table 2:** Lowest detection limit of cytokines, growth factors and others in cytokine bead array


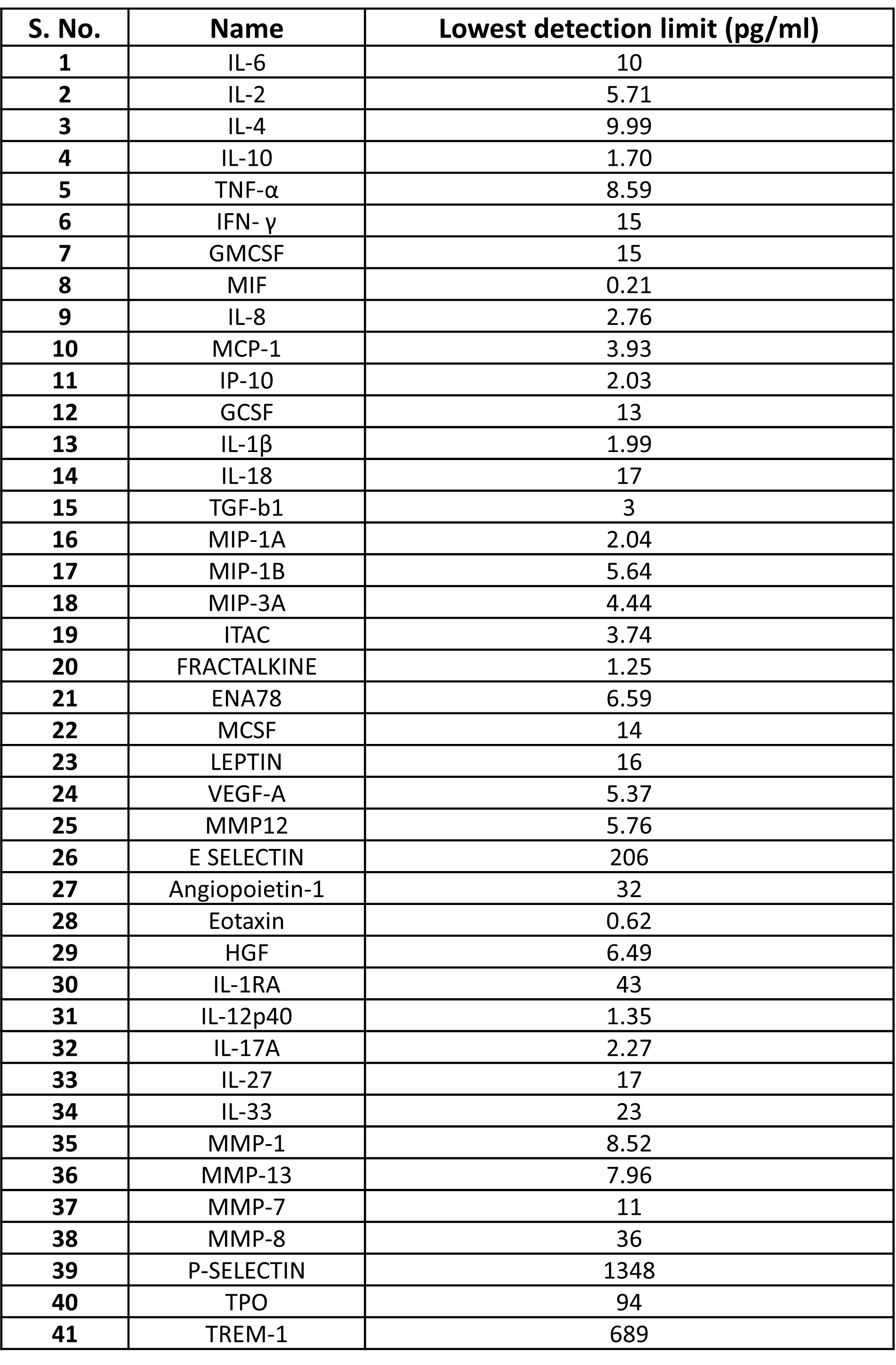


**Supplementary Table 3:** List of primer sequences for qRT-PCR.


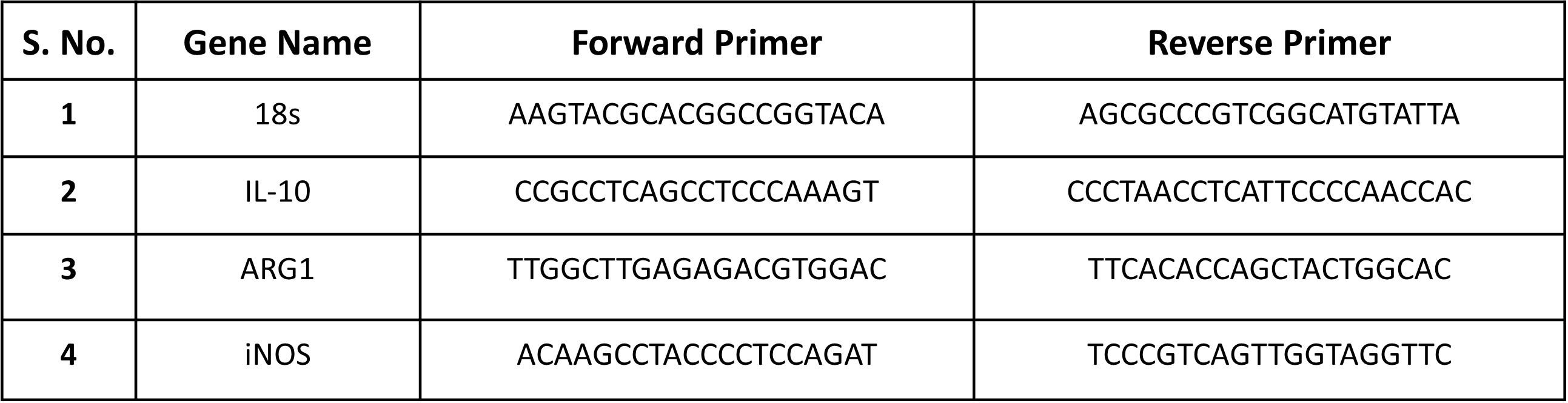


**Supplementary Table 4:** Patient characteristics of two Sepsis patient groups; without GM-CSF treatment and with GM-CSF treatment with Day 1 and Day 3 follow-up


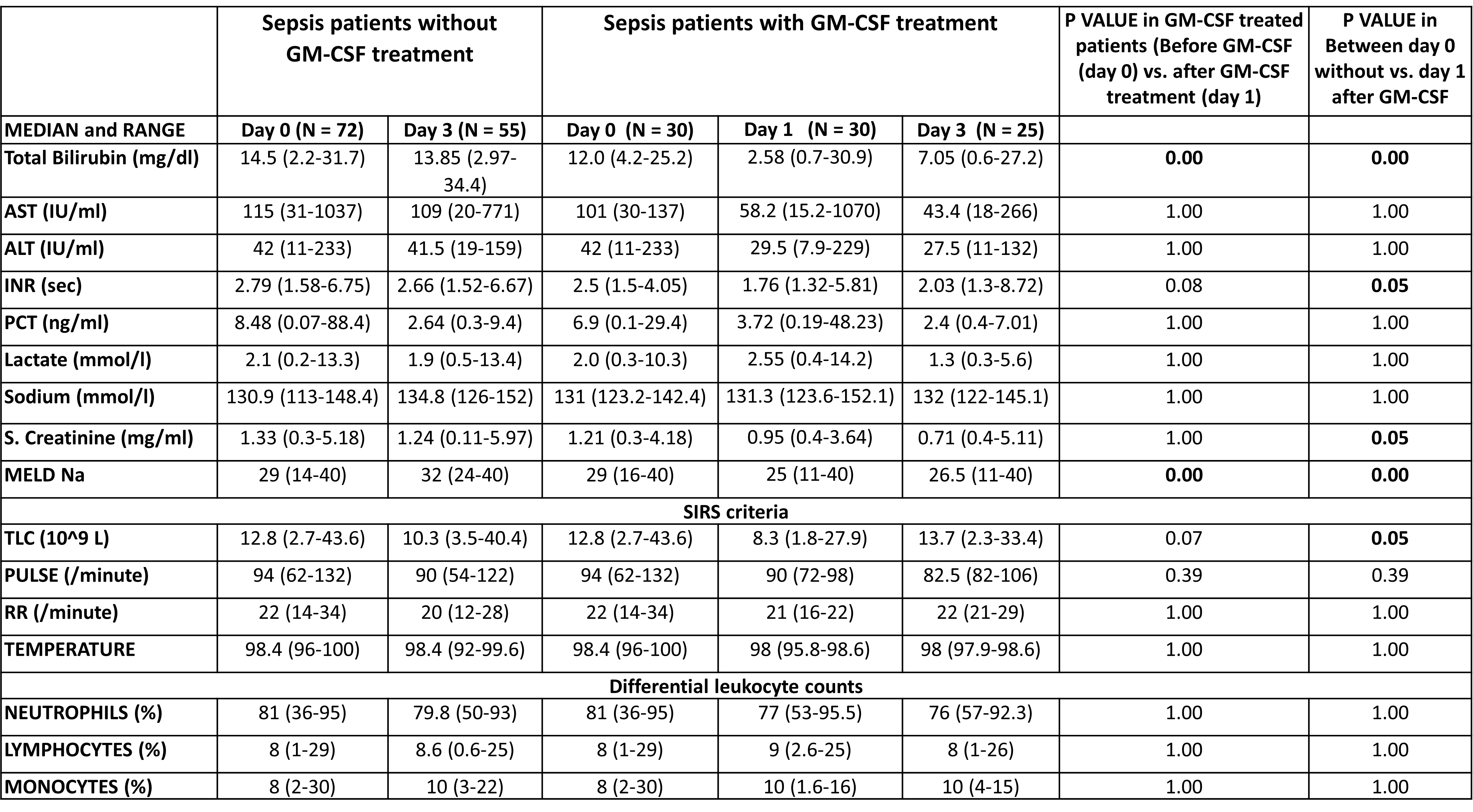


**SUPPLEMENTARY FIGURES:**

**Supplementary Figure 1:** Study cohort and study design

**
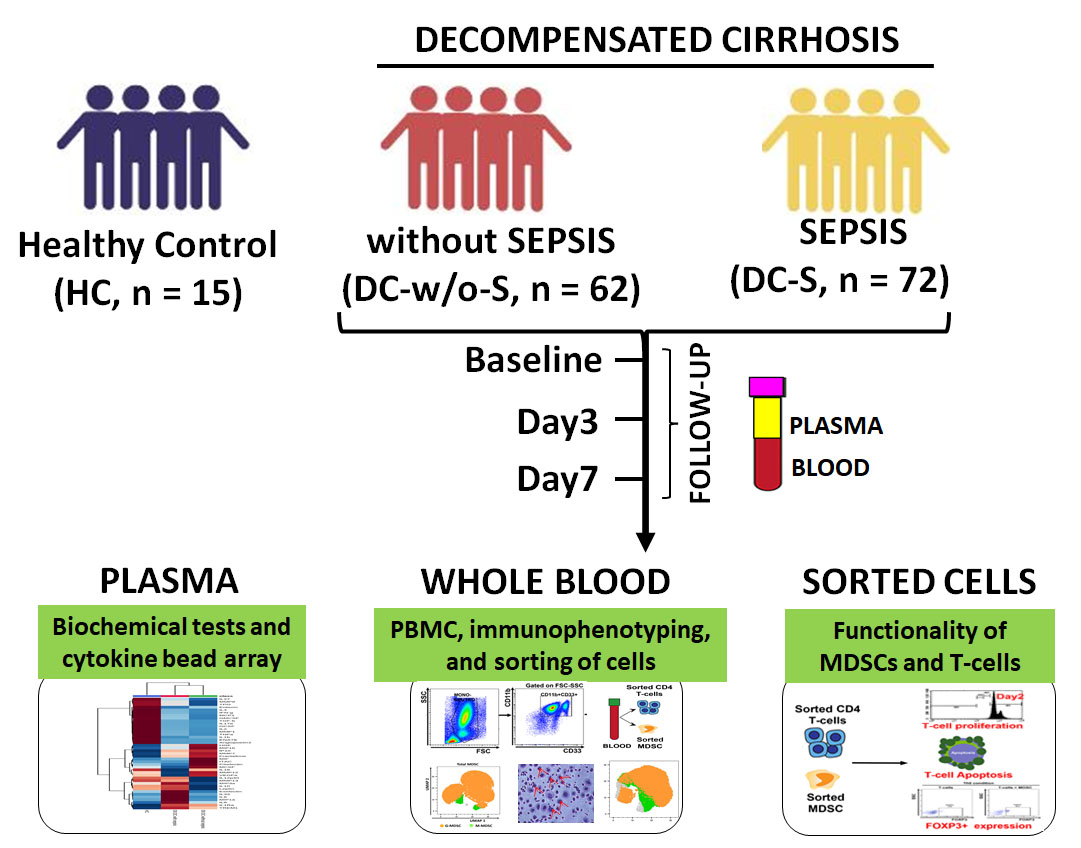
**

**Results: Immune scan**

With high dimensional flow cytometry, whole blood immune scan was analyzed for each cell type. Total lymphocyte frequencies were decreased reflecting lymphopenia while neutrophil frequencies were increased reflecting neutrophilia in sepsis patients compared to HC as it coincides with the differential leukocyte count (DLC) of cells of the study groups. No significant difference observed in lymphocytes and monocytes on follow ups, but neutrophils decreased at day7 compared to day0 in sepsis patients


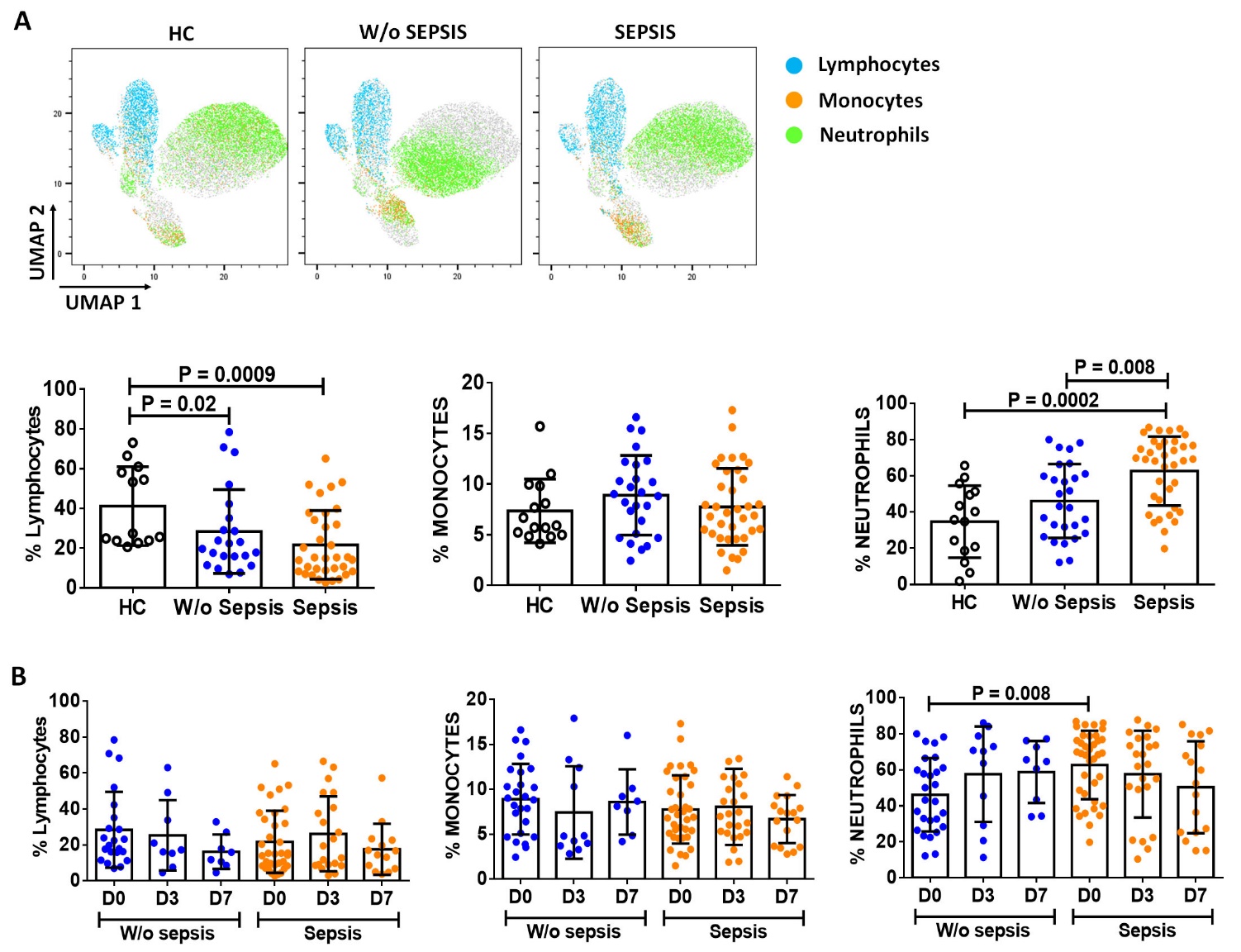


**Supplementary Figure 2: Expression of lymphocytes, monocytes and neutrophils** **A)** UMAP visualization of pooled immune cells i.e lymphocytes (light blue color), monocytes (orange color) and neutrophils (green color) and scatter dot plot shows %frequency of lymphocytes, monocytes and neutrophils in HC (black color), w/o sepsis (blue color) and sepsis patients. **B)** Scatter dot plot shows %frequency of lymphocytes, monocytes and neutrophils at D0, D3 and D7 in w/o and with sepsis group. Results are expressed as the mean ± SD; a-b) One-way ANOVA/ Kruskal–Wallis test followed by probability adjustment by the Mann–Whitney.

**Supplementary Figure 3: A)** Comparison of gating strategies by Agrati et al. Cell Death Differ (2020) and Pallet et al. Nat Med. 2015, **B)** %frequency of MDSCs, G-MDSCs and M-MDSCs in sepsis (orange color) and w/o sepsis (blue color) patients at D0, D3 and D7. Results are expressed as the mean ± SD; One-way ANOVA/ Kruskal–Wallis test followed by probability adjustment by the Mann–Whitney.


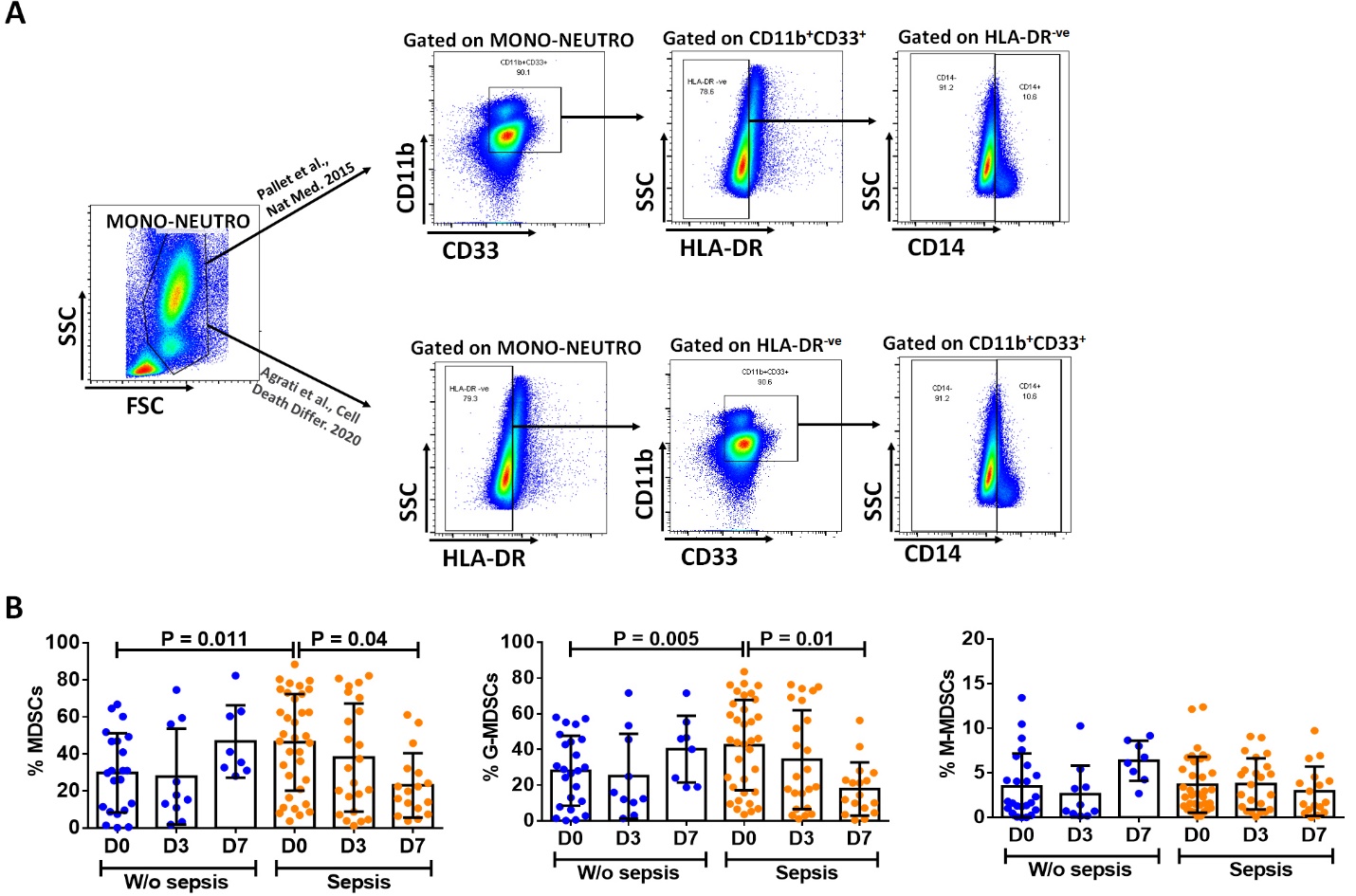


**Supplementary Figure 4:** Scatter dot plot shows %frequency of **A)** %CD4 T-cells, and **B)** CD4 subsets i.e T_CM_, T_NAIVE_, T_EM_, T_EMRA_ in sepsis (blue color) and w/o sepsis patients (orange color) at D0, D3 and D7. **C-D)** Gating strategy for the expression of Tregs at D0, D3 and D7. Results are expressed as the mean ± SD; a,b and d) One-way ANOVA/ Kruskal–Wallis test followed by probability adjustment by the Mann–Whitney.


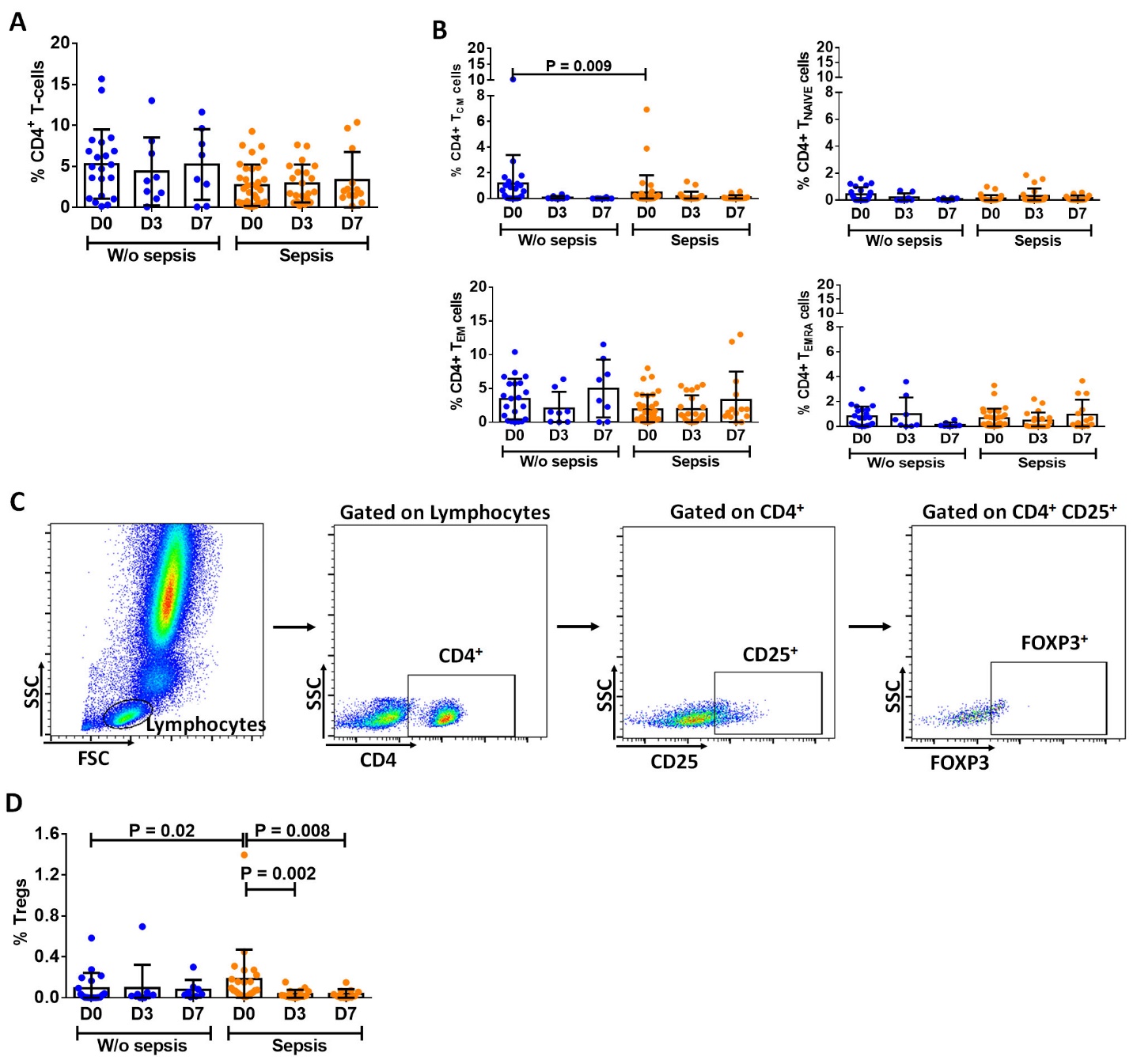


**Supplementary Figure 5: Cytokines and growth factors linked with MDSCs. A)** Plasma levels of cytokines and growth factors (pg/ml) related to MDSCs in HC, without sepsis and with sepsis. **B)** Heatmap showing expression of MDSCs related plasma cytokines and growth factors in between the groups. **C)** Correlation matrix depicting the correlation between cytokines and MDSCs. Correlation matrix was prepared using "corrplot" R package and can be visualized using a correlogram. Red color represents positive correlation and green color represents negative correlation.


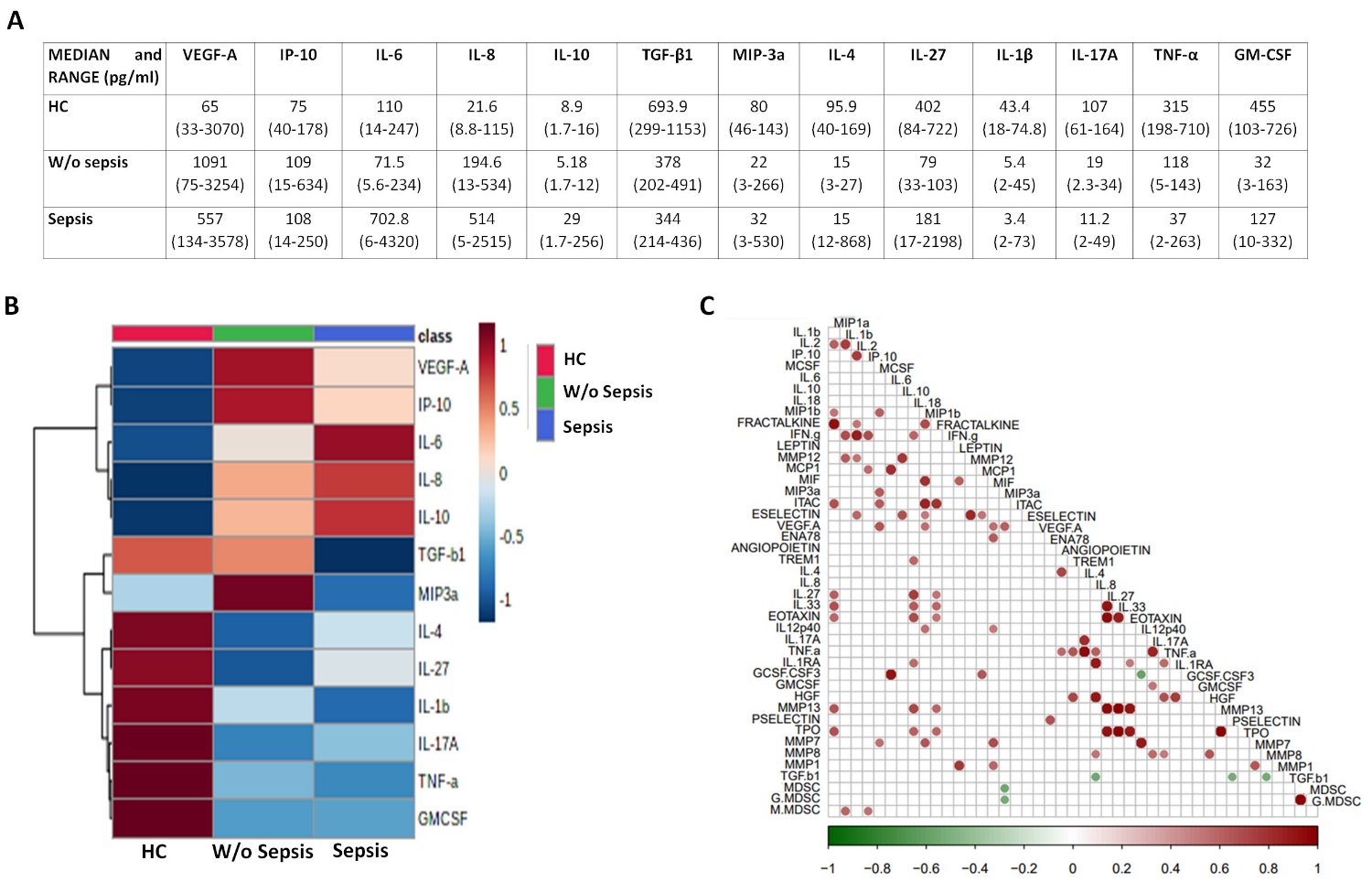


**Supplementary Figure 6:** Representative experimental scheme used for **A)** T-cell apoptosis and **B)** T-cell proliferation.


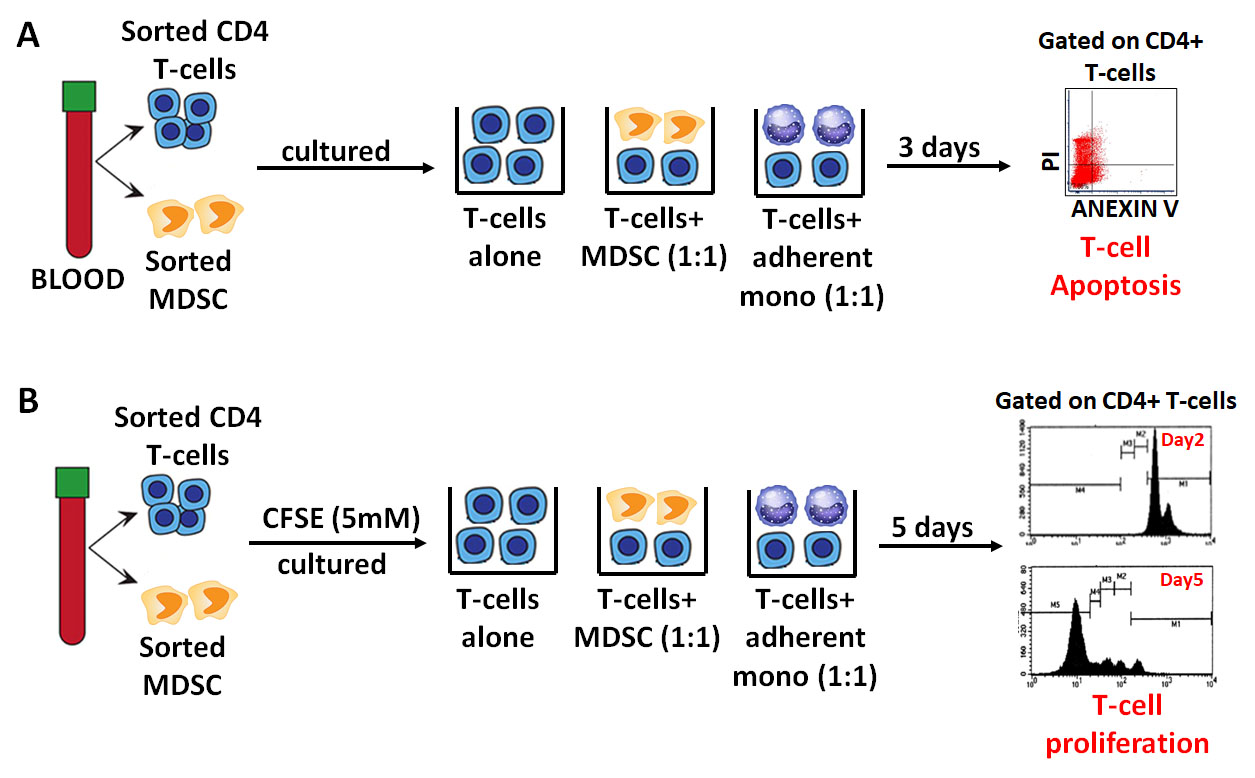


**Supplementary Figure 7: A)** Representative CFSE proliferation. Bar diagrams shows **B)** %Apoptosis and **C)** %proliferation at different time points in w/o and with sepsis group in T-cells cultured alone (black color), with MDSCs (white color) and with monocytes (grey color). Results are expressed as the mean ± SD; Mann–Whitney/t-test within the patient groups,


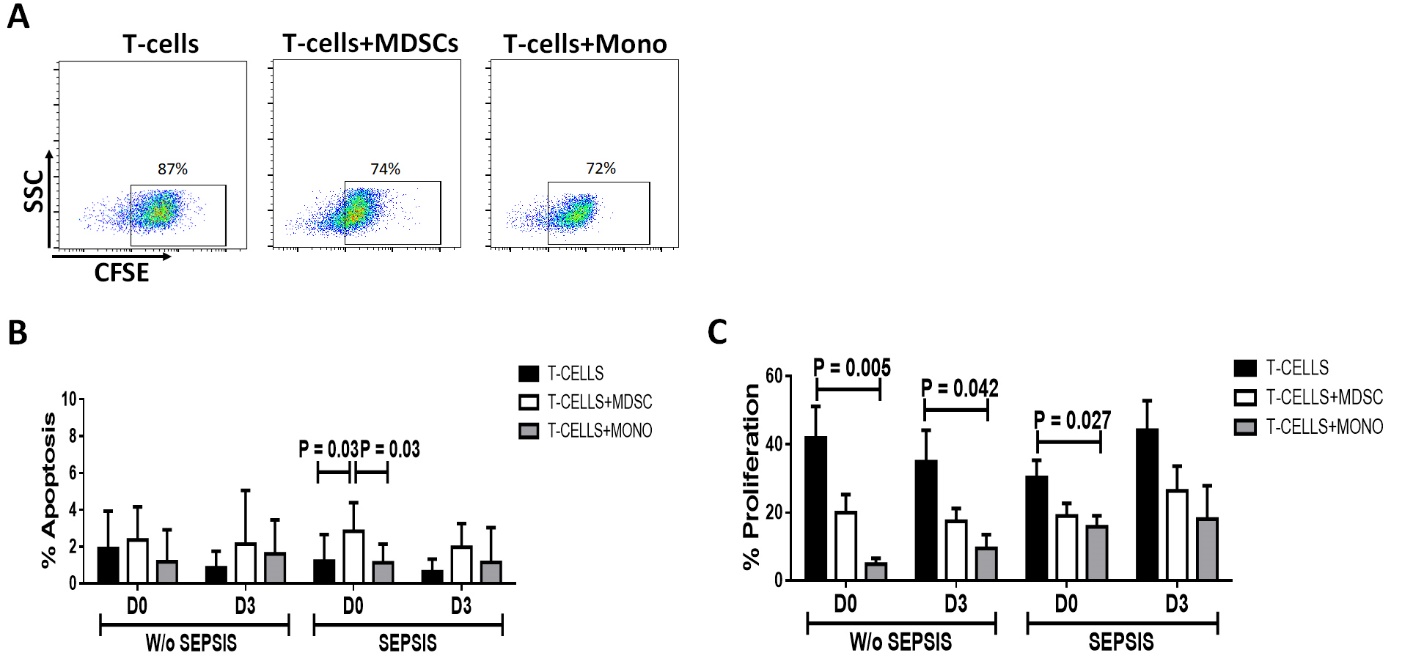


**Supplementary Figure 8:** Bar diagrams showing expression of % FOXP3^+^ on CD4^+^ T-cells in **A)** TH0, and **B-C)** TH17 condition in T-cells cultured alone (black color), and with MDSCs (white color) at different time points. **D)** Expression of %FOXP3^+^ on CD4^+^ T-cells cultured with MDSCs and in stimulations with rTGF-β, L-NMMA and nor-NOHA in w/o sepsis (grey color), and sepsis (white color) at different time points. Results are expressed as the mean ± SD; a-c) Mann–Whitney/t-test within the patient groups, and d) Kruskal–Wallis test within the group along with the multiple comparisons.


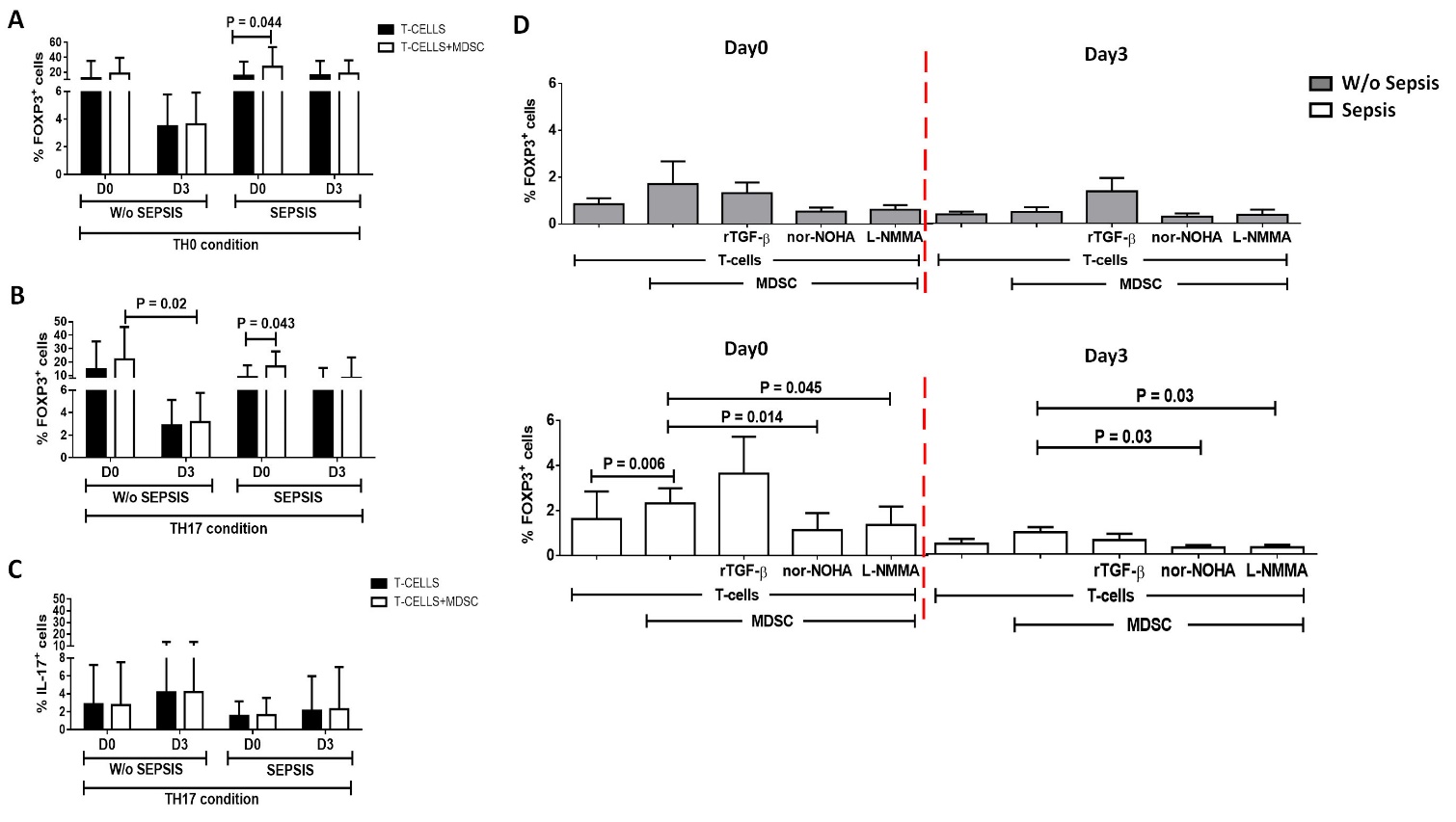


**Supplementary Figure 9:** Scatter dot plots show the expression of **A)** %HLA-DR on monocytes and %CD11b^+^CXCR1^+^ cells on Neutrophils and **B)** %frequency of G-MDSCs and M-MDSCs in sepsis patient without (blue color) and with GM-CSF (orange color) at different time points. Results are expressed as the mean ± SD; One-way ANOVA/ Kruskal–Wallis test followed by probability adjustment by the Mann–Whitney.

**
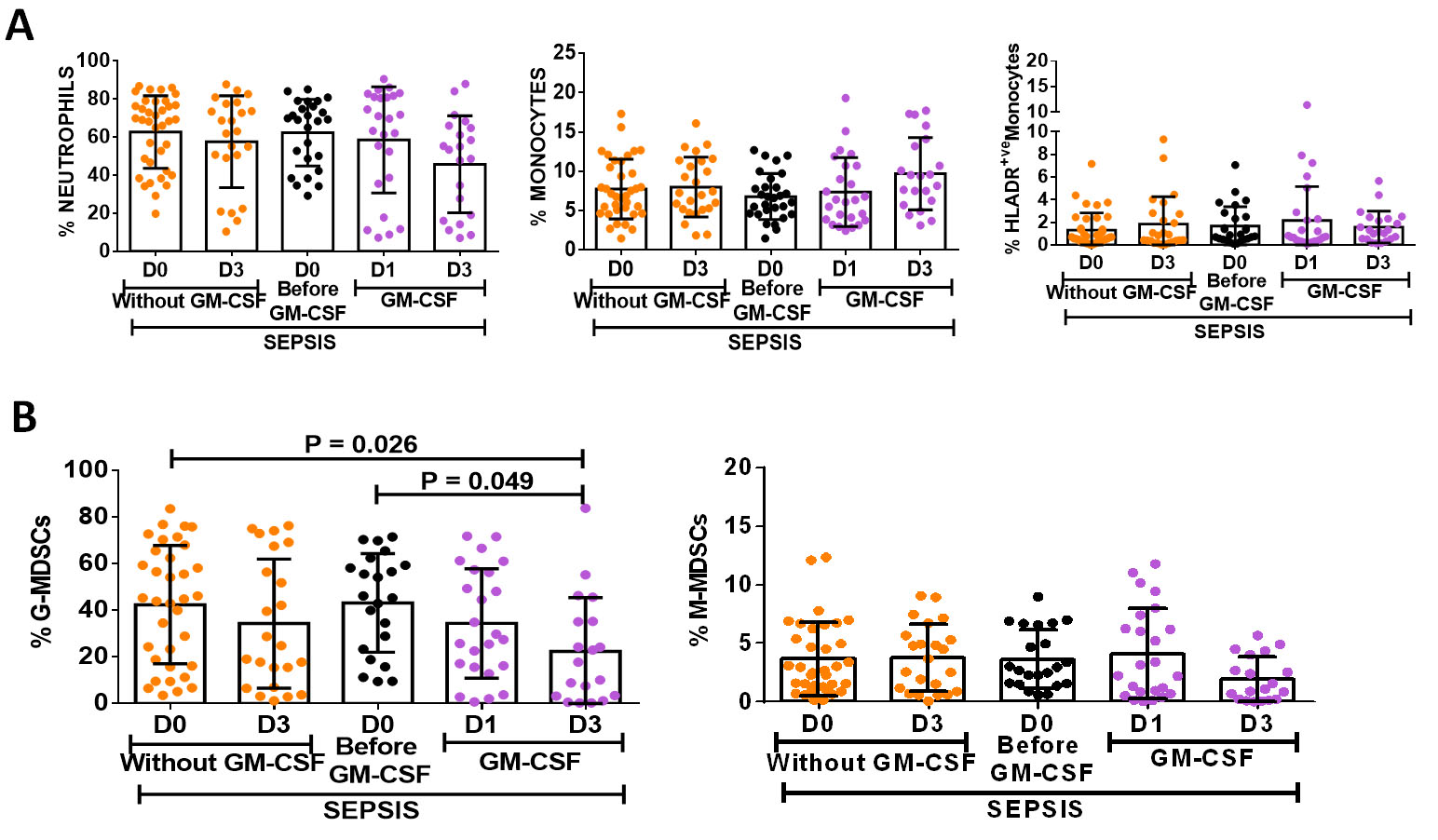
**
